# UBR5 forms ligand-dependent complexes on chromatin to regulate nuclear hormone receptor stability

**DOI:** 10.1016/j.molcel.2023.06.028

**Published:** 2023-07-20

**Authors:** Jonathan M. Tsai, Jacob D. Aguirre, Yen-Der Li, Jared Brown, Vivian Focht, Lukas Kater, Georg Kempf, Brittany Sandoval, Stefan Schmitt, Justine C. Rutter, Pius Galli, Colby R. Sandate, Jevon A. Cutler, Charles Zou, Katherine A. Donovan, Ryan J. Lumpkin, Simone Cavadini, Paul M.C. Park, Quinlan Sievers, Charlie Hatton, Elizabeth Ener, Brandon D. Regalado, Micah T. Sperling, Miko1aj S1abicki, Jeonghyeon Kim, Rebecca Zon, Zinan Zhang, Peter G. Miller, Roger Belizaire, Adam S. Sperling, Eric S. Fischer, Rafael Irizarry, Scott A. Armstrong, Nicolas H. Thomä, Benjamin L. Ebert

**Affiliations:** 1 Department of Pathology, Brigham and Women’s Hospital, Harvard Medical School, Boston, MA, USA; 2 Division of Oncology, Dana-Farber Cancer Institute, Harvard Medical School, Boston, MA, USA; 3 Broad Institute of MIT and Harvard, Cambridge, MA, USA; 4 Friedrich Miescher Institute for Biomedical Research, Basel, Switzerland; 5 Department of Molecular & Cellular Biology, Harvard University, Cambridge, MA, USA; 6 Department of Data Science, Dana-Farber Cancer Institute, Harvard Medical School, Boston, MA, USA; 7 Faculty of Science, University of Basel, Basel, Switzerland; 8 Department of Cancer Biology, Dana-Farber Cancer Institute, Boston, MA, USA; 9 Department of Biological Chemistry & Molecular Pharmacology, Harvard Medical School, Boston, MA, USA; 10 Pediatric Hematology-Oncology, Boston Children’s Hospital, Dana-Farber Cancer Institute, Harvard Medical School, Boston, MA, USA; 11 Center for Cancer Research, Massachusetts General Hospital, Boston, MA, USA; 12 Division of Pathology, Dana-Farber Cancer Institute, Harvard Medical School, Boston, MA, USA; 13 Division of Hematology, Department of Medicine, Brigham and Women’s Hospital, Harvard Medical School, Boston, MA, USA; 14 Howard Hughes Medical Institute, Boston, MA, USA; 15 These authors contributed equally; 16 Lead contact

## Abstract

Nuclear hormone receptors (NRs) are ligand-binding transcription factors that are widely targeted therapeutically. Agonist binding triggers NR activation and subsequent degradation by unknown ligand-dependent ubiquitin ligase machinery. NR degradation is critical for therapeutic efficacy in malignancies that are driven by retinoic acid and estrogen receptors. Here, we demonstrate the ubiquitin ligase UBR5 drives degradation of multiple agonist-bound NRs, including the retinoic acid receptor alpha (RARA), retinoid x receptor alpha (RXRA), glucocorticoid, estrogen, liver-X, progesterone, and vitamin D receptors. We present the high-resolution cryo-EMstructure of full-length human UBR5 and a negative stain model representing its interaction with RARA/RXRA. Agonist ligands induce sequential, mutually exclusive recruitment of nuclear coactivators (NCOAs) and UBR5 to chromatin to regulate transcriptional networks. Other pharmacological ligands such as selective estrogen receptor degraders (SERDs) degrade their receptors through differential recruitment of UBR5 or RNF111. We establish the UBR5 transcriptional regulatory hub as a common mediator and regulator of NR-induced transcription.

## In brief

Tsai, Aguirre et al. identify an E3 ubiquitin ligase, UBR5, that ubiquitylates a large subset of nuclear hormone receptors through direct competition with nuclear coactivators, resulting in their degradation. UBR5 adopts a giant tetrameric ring-shaped architecture that is recruited to agonist-bound NRs directly on chromatin to regulate transcription.

## Graphical abstract

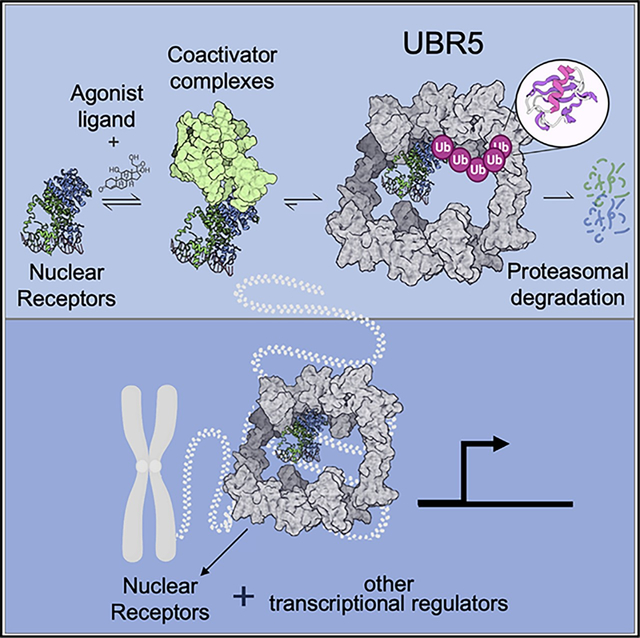

## INTRODUCTION

Nuclear hormone receptors (NRs) are a family of ~48 transcription factors that bind a range of hydrophobic steroids, hormones, and fatty acid ligands, whereas additional NRs, termed orphan receptors, do not have known ligands. Perturbations of NR activity drive a range of cancers, fibroses, inflammatory conditions, and metabolic disorders.^1–4^ Upon binding to NRs, canonical agonist ligands recruit nuclear coactivator (NCOA) proteins and transcription machinery to induce target gene expression, while antagonist ligands suppress gene expression through recruitment of repressive complexes.^5^ In addition to activating transcription, many agonist ligands promote the subsequent degradation of their cognate NRs, which has been described for the retinoic acid receptor alpha (RARA),^6^ thyroid hormone receptor (TR),^7^ glucocorticoid receptor (GR),^8^ and estrogen receptor (ER)^9^ among others.^1^

NR ligands are widely used and highly effective drugs, and NR degradation is an important feature of therapeutic activity. NR degradation is critical for the activity of the endogenous RARA agonist, all-*trans* RA (ATRA), in the treatment of acute promyelocytic leukemia (APML), driven by a fusion of the *RARA* and the promyelocytic protein (*PML*) genes.^10^ Cure of APML requires both the activation of transcriptional programs by PML-RARA and degradation of the fusion protein in response to ATRA and arsenic trioxide (ATO).^11–15^ Selective ER degraders (SERDs), such as fulvestrant, are widely used in the treatment of breast cancer, and novel therapies that induce degradation of ER are in clinical trials.^16–20^ The ubiquitin-proteasome system (UPS) has been implicated in these degradation processes, but the mechanism and factors responsible are not well characterized.

NRs are structurally related, each containing a DNA-binding domain (DBD) and a ligand-binding domain (LBD). The LBD contains a conserved hydrophobic cleft that permits binding by a common set of NCOAs and a variable ligand-binding pocket that accommodates structurally diverse ligands.^21^ Given their structural similarities, it is conceivable that a common degradation mechanism regulates a broad range of NRs. Here, we used genetic screens to identify the HECT-family protein ubiquitin protein ligase E3 component n-recognin 5 (UBR5) as the E3 ubiquitin ligase that governs agonist-induced degradation of numerous NRs and characterized these interactions biochemically and structurally. Our studies reveal that UBR5 recognizes a common degron that also engages NCOAs, leading to inherent competition on chromatin between NR activation and degradation processes.

## RESULTS

### CRISPR screens identify UBR5 as a principal ligand-dependent regulator of NR degradation

**Figure 1.**
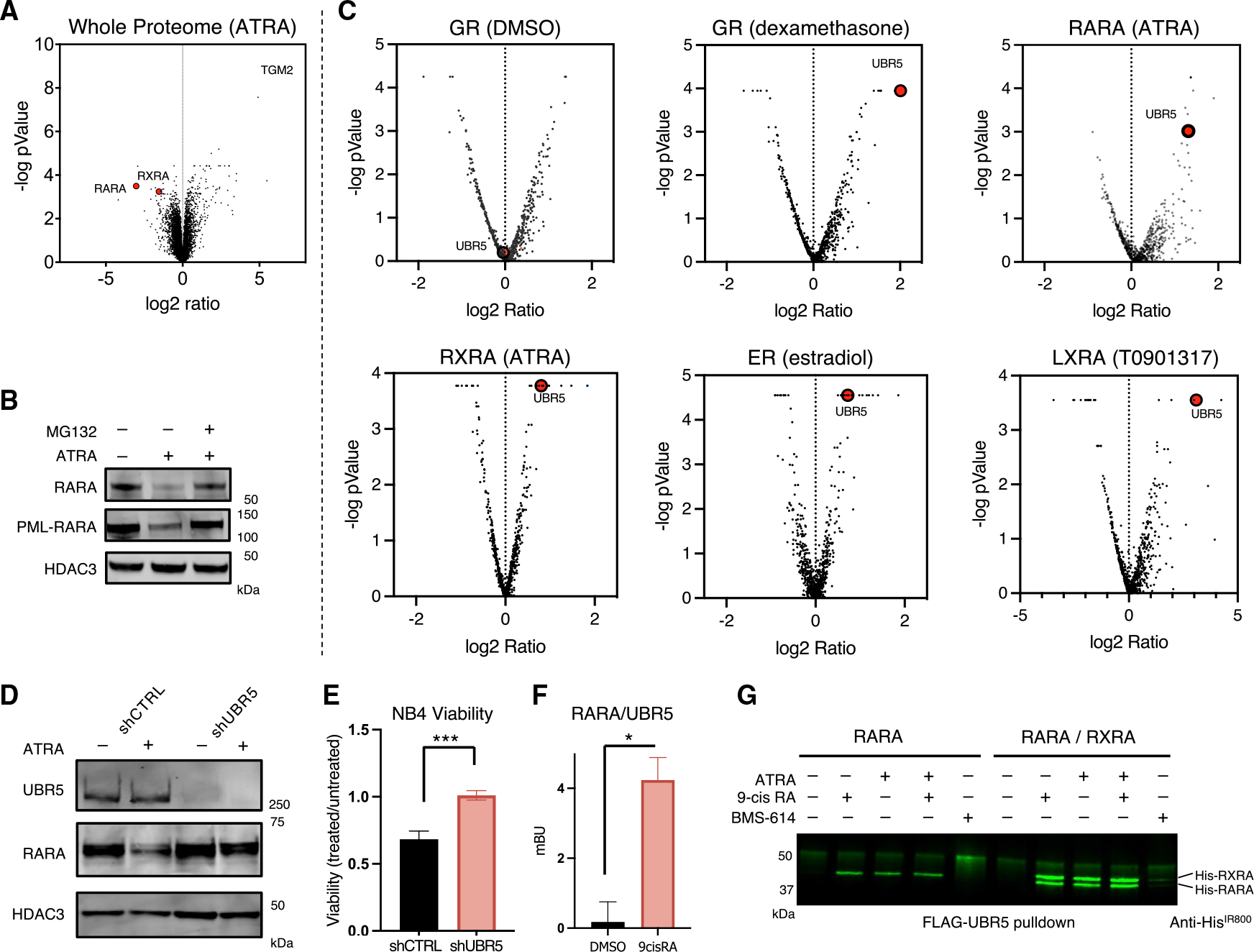
CRISPR screens identify UBR5 as a principal ligand-dependent regulator of NR degradation (A) Whole proteomics of NB4 cells treated with 10 mM ATRA for 24 h (n = 3 per condition). (B) Western blotting of PML-RARA, RARA, and HDAC3 in NB4 cells following treatment with ATRA and proteasome inhibitor (MG132). (C) Volcano plots of targeted CRISPR screens highlighting enrichment of UBR5 following ligand treatment of indicated NR fluorescent reporter U937 cell lines. UBR5 is highlighted in red. (D) Western blotting of NB4 cells transduced with shRNAs against luciferase (shCTRL) or UBR5 treated with or without ATRA. (E) Viability by relative cell counts of NB4 cells transduced with control or UBR5 shRNAs (two shRNAs per condition, combined) following treatment with 1 μM ATRA for 24 h. (n = 4, two-sided t test). (F) Bioluminescence resonance energy transfer (BRET) assay of RARA and UBR5, treated ± 9-*cis* RA. (n = 3 two-sided t test) (G) Western blot showing *in vitro* FLAG-UBR5 pull-down of purified His-tagged RARA or RARA/RXRA heterodimer in the presence of 50 μM specified retinoid agonist or antagonist. (NS, not significant, *p < 0.05, **p < 0.005, ***p < 0.0005, ****p < 0.00005, error bars represent SEM.)

To investigate NR protein stability, we first interrogated RARA, a well-studied hormone receptor whose degradation is necessary for the efficacy of leukemia therapy.^15^ Whole-proteome mass spectrometry analysis revealed that protein levels of RARA and its heterodimeric partner, retinoid X receptor alpha (RXRA), were significantly and selectively decreased following 24 h of ATRA treatment (Figures 1A and S1A). RARA and PML-RARA degradation was dependent on the proteasome, but not neddylation, suggesting that degradation is mediated by a ubiquitin ligase not belonging to the cullin-RING family (Figures 1B and S1B).

Next, we generated fluorescent reporters containing the full-length coding sequence for five NRs, which are known to be degraded by their cognate ligands: GR, RARA, RXRA, ER, and liver-X receptor alpha (LXRA), fused in-frame with eGFP followed by an internal ribosome entry site (IRES) and mCherry for signal normalization (Figure S1C). Ligand-dependent reporter degradation was confirmed for each receptor and RARA reporter degradation was similarly proteasome dependent and neddylation independent (Figures S1D–S1F). To identify the specific enzymatic components of the UPS involved in NR degradation, we performed a CRISPR-Cas9 screen on our NR reporter lines using an sgRNA library targeting ~700 E3 ubiquitin ligases, E2 conjugating enzymes, and deubiquitinating enzymes (Figure S1G).^22,23^ Strikingly, sgRNAs targeting the E3 ligase UBR5 significantly prevented degradation of all five NR reporters following treatment with their ligands and was enriched over cells treated with DMSO (Figures 1C and S1H).

Because *UBR5* is a common essential gene, and sustained *UBR5* knockout using sgRNAs is lethal to cultured cells,^24^ we used RNA interference to decrease *UBR5* expression and validate UBR5 as a regulator of ATRA-induced degradation of RARA. Knockdown of *UBR5* in the APML cell line NB4, rescued ATRA-induced RARA degradation, as assessed by western blot, compared with cells expressing control shRNAs (Figures 1D and S1I).

PML-RARA is degraded therapeutically by two small molecules: ATRA and ATO, which target RARA and PML, respectively.^6,11^ We found that shRNA-mediated knockdown of *UBR5* prevented ATRA-induced degradation of PML-RARA in NB4 cells but did not alter the effects of ATO (Figure S1J). Moreover, knockdown of *UBR5* decreased ATRA sensitivity in NB4 cells (Figures 1E and S1K), indicating that UBR5 is required for the efficacy of ATRA in APML treatment.

### RARA recruits UBR5 in an agonist-dependent manner

To assess UBR5 and RARA co-localization in cells, we performed a bioluminescence resonance energy transfer (BRET) assay. Cells treated with RA exhibited more luminescence than controls (Figure 1F), showing that UBR5/RARA co-localization is ligand-dependent *in vivo*.

To confirm that UBR5 and RARA interact directly, we examined binding of the purified recombinant proteins *in vitro*. Full-length human FLAG-UBR5 was purified from transiently transfected HEK293 cells, immobilized on FLAG M2 beads, and assayed for RARA binding in the presence or absence of retinoids. A stark ligand dependence was observed for UBR5-RARA complex formation in the presence of RARA agonists; ATRA, 9-*cis* RA, or an equimolar mixture of both, robustly induced binding of RARA to UBR5 (Figure 1G). Conversely, the RARA-selective antagonist, BMS614, prevented complex formation. In cells, RARA activates transcription as a heterodimer with RXRA.^25^ We therefore tested whether RARA would still be recruited to UBR5 in a heterodimeric complex with RXRA. Indeed, a RARA/RXRA heterodimer robustly bound to UBR5 *in vitro* in the presence of both agonists and, notably, appeared to be a better substrate for UBR5 binding compared with RARA alone. UBR5-RARA/RXRA complex formation, on the other hand, was similarly hindered by the RARA antagonist BMS614 (Figure 1G). Taken together, the results show that RARA/RXRA binds UBR5 *in vivo* and *in vitro*, and that complex formation is induced when these NRs are in an agonist-bound state.

### UBR5 competes with NCOAs for the conserved hydrophobic cleft of NRs

To identify the degron recognized by UBR5, we performed deletion mapping using our RARA reporter. The RARA LBD bound UBR5 *in vitro* (Figure 1G) and still mediated ATRA-induced reporter degradation (Figure S2A), indicating the RARA LBD is sufficient for recognition by UBR5. Further truncations into the LBD no longer sustained degradation following ATRA treatment.

**Figure 2.**
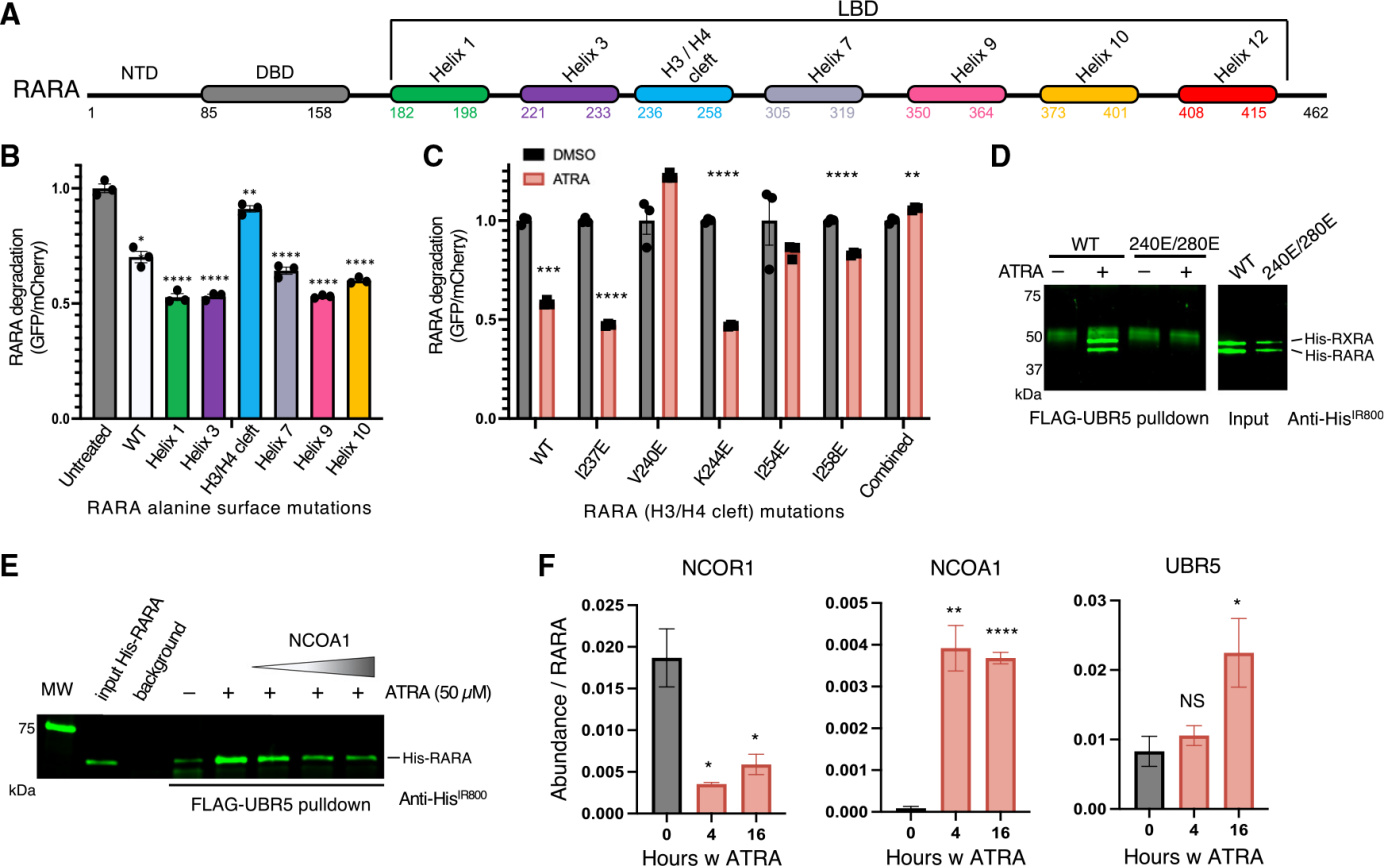
UBR5 competes with nuclear coactivators for the conserved hydrophobic cleft of NRs (A) Schematic of RARA domain architecture; NTD, N-terminal domain; DBD, DNA-binding domain; LBD, ligand-binding domain. (B) Degradation of RARA reporters containing 4–5 surface-exposed alanine mutations per helix, relative to untreated (DMSO) (n = 3 two-sided t test). See Figure S2 for detailed amino acid substitutions. (C) Degradation of RARA reporters containing glutamate-substituted residues within the H3/H4 hydrophobic cleft. (n = 3 two-sided t test). (D) Western blot showing *in vitro* FLAG-UBR5 pull-down of purified His-tagged RARA/RXRA heterodimer, with or without single residue substitutions in the hydrophobic cleft and with or without 50 μM ATRA. (E) Competitive nature of RARA binding to UBR5 and NCOA1 demonstrated by UBR5 pull-down with purified proteins. FLAG-UBR5 (15 pmol) was pre-bound to a large excess of His-RARA in the presence of ATRA and excess RARA protein washed away. Increasing amounts of full-length NCOA1 protein were then added to outcompete the UBR5-RARA interaction. NCOA was titrated with a 0.5, 5, and 10× molar excess relative to UBR5. (F) Relative abundances in RARA-GFP IP-MS of NCOR1, NCOA1, and UBR5 normalized to RARA abundance following 0, 4, and 16 h of ATRA treatment. (NS, not significant, *p < 0.05, **p < 0.005, ***p < 0.0005, ****p < 0.00005, error bars are SEM.)

Ligand binding induces a stereotypic change in the NR LBD: Helix 12 rotates to cover the ligand pocket and exposes a conserved hydrophobic cleft between helices H3 and H4 used for NCOA binding.^26^ To identify a degron required for recognition by UBR5, we systematically mutated solvent exposed residues in the RARA LBD to alanine. Only substitutions within the hydrophobic cleft prevented ATRA-induced degradation (Figures 2B, S2B, and S2C). To further perturb these hydrophobic interactions, we mutated residues in the hydrophobic cleft to glutamate. Mutations of I254 and I258 significantly reduced ATRA-induced degradation, and a single mutation of V240 in RARA abolished ATRA-induced degradation (Figure 2C). Mutating the corresponding cleft residues in RXRA (V280, equivalent to RARA V240), similarly abrogated RXRA degradation (Figure S2D). These residues, although critical for substrate degradation, were previously reported to retain ligand binding in several NRs.^27,28^

Having demonstrated that mutations of V240 in RARA and V280 in RXRA impair ligand-induced reporter degradation, we examined whether these residues are critical for UBR5 binding. RARA and RXRA proteins were purified containing single glutamate substitutions at V240 and V280, respectively, and these abolished agonist-induced binding to UBR5 *in vitro* (Figure 2D). Moreover, the equivalent single-residue substitutions blocked ATRA-induced degradation of the PML-RARA-GFP reporter in cells (Figure S2E). These findings define the H3/H4 hydrophobic cleft as the NR interface required for RARA degradation and rationalize the agonist-dependence of UBR5 association.

NCOAs, likewise, bind the hydrophobic cleft of NRs through one or more Leu-X-X-Leu-Leu (LxxLL) motifs following ligand binding. This suggested that NCOA and UBR5 engagement could be mutually exclusive. In agreement with this hypothesis, we found UBR5 binding to RARA *in vitro* was impaired by an excess of full-length NCOA1 (Figure 2E), confirming that NCOA1 and UBR5 directly compete for the H3/H4 hydrophobic cleft. To examine the temporal dynamics of this competition in cells, we performed sequential RARA immunoprecipitation/mass spectrometry (IP/MS) in the presence of MG132 at 0, 4, and 16 h following ATRA treatment. The repressive complex (including NCOR1 and NCOR2) associated with RARA in the absence of ATRA, and was replaced by the NCOAs (NCOA1, NCOA2, and NCOA3) and the mediator complex at subsequent time points with ATRA treatment (Figures 2F and S2F). We contemporaneously observed an enrichment of UBR5 peptides at 16 h post ATRA treatment. Similar findings were observed by IP-western blot, with initial NCOA1 binding to RARA after 4 h of ATRA treatment, diminishing NCOA1 binding from 4 to 16 h, and increased binding of UBR5 at 16 and 24 h, coinciding with RARA degradation (Figure S2G). Taken together, these data suggest that NCOAs engage NRs following a hormone signal and are subsequently outcompeted by UBR5, leading to NR degradation.

### Structural and functional characterization of UBR5

**Figure 3.**
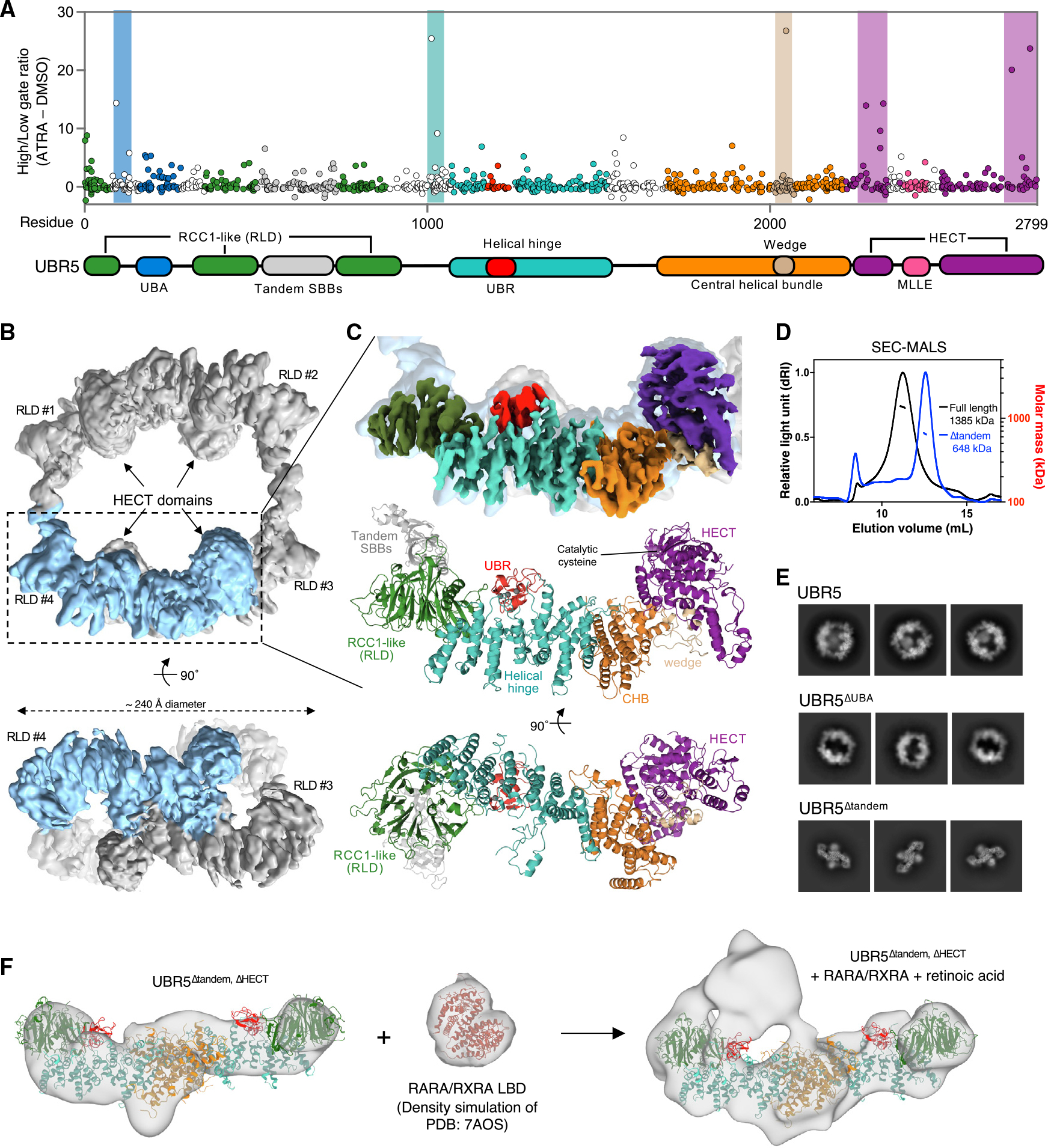
Structural and functional characterization of UBR5 (A) UBR5 tiling screen of RARA-GFP reporter degradation with domain map shown beneath according to residue numbers. Highlighted residues represent guides with over 10-fold enrichment. (B) Cryo-EM density map of UBR5 tetramer, with one monomeric unit colored in light blue, and shown rotated by 90° beneath. (C) Expanded view of the dashed box outlined in (B), showing a surface map at the higher resolution obtained in the dimeric UBR5 map, with domains colored according to the domain architecture above. A cartoon representation of the UBR5 model in the same orientation is shown below. The tandem SBB structure predicted by AlphaFold2 is included for completeness in light gray. (D) Size-exclusion multiple-angle light scattering (SEC-MALS) of full-length and Δtandem UBR5 variant, showing how removal of the tandem SBBs changes the assembly from tetramer to dimer. (E) 2D-class averages for indicated UBR5 variants. Deletion of residues comprising the UBA insertion led to a loss of density in the middle of the ring, whereas deletion of residues comprising the tandem domain led to a dimeric species of UBR5 with altered preferred orientation. Each class represents ~3,000 unique particles. (F) EM density maps of indicated complexes obtained by negative stain EM, limited by resolution to >20 Å. A simulated density map of RARA/RXRA generated by molmap (ChimeraX) and low-pass filtered to 25 Å is shown in center as a reference for the expected additional density size.

To identify the domains in UBR5 that govern hormone-dependent NR degradation, we performed a sgRNA CRISPR tiling screen.^29^ A UBR5 tiling library with ~1,660 sgRNAs was transduced into RARA-GFP reporter cells, treated with ATRA or DMSO, and analyzed to identify sgRNAs that prevent ATRA-induced RARA degradation. The screen revealed multiple *UBR5* regions required for ATRA-induced RARA degradation, including the N-terminal ubiquitin-associating (UBA) domain, C-terminal HECT domain, as well as central regions contained within the helical hinge and central helical bundle (Figure 3A).

We next structurally interrogated functional domains in the context of full-length UBR5, which is expressed predominantly as a 310 kDa polypeptide product in humans. Although small individual domains (UBA, HECT, and MLLE) have been resolved^30–32^ the overall architecture of UBR5 remains unknown. We determined the structure of full-length UBR5 by cryo-EM, which, following further classification and refinement, reached ~3.9 Å resolution (Figures 3B, 3C, and S3; Table S1). Intriguingly, 2D class averages and the reconstructed 3D volumes showed a ring-like structure with distinguishable symmetric features (Figure 3E). Size-exclusion multi-angle light scattering (SEC-MALS) identified a main species with a molecular weight of 1.385 MDa, consistent with a tetrameric assembly (Figure 3D).

The overall architecture of UBR5 is a closed and twisted ring with dihedral (D2) symmetry, held together by two distinct dimerization interfaces. One dimerization interface is maintained through a large central helical bundle (residues 1,350–2,110), which is among the highest resolution regions of the EM map. A second dimerization interface holds together two halves of the ring and is markedly more flexible. To unambiguously assign this weaker density, we expressed and purified a mutant version of UBR5 lacking residues 522–720 (UBR5^Δtandem^), which in SEC-MALS was consistent with a dimer (648 kDa) (Figure 3D). In cryo-EM analysis the dimeric UBR5^Δtandem^ is held together solely by the rigid interface in the central helical bundle (Figures 3E, bottom, S4A, and S4B). Reduced orientation bias of the dimeric UBR5 particles allowed us to generate an improved map at ~3.4 Å resolution.

The N-terminal region of UBR5 arranges into a seven-bladed β-propeller, which shows weak homology to a regulator of chromosome condensation 1 (RCC1)-like domain (RLD). Unlike most RLDs, however, the beta-propeller in UBR5 contains two large insertions following the 2^nd^ and 5^th^ propeller blades. The insertion following the 2^nd^ blade is largely unobserved in our EM reconstructions and is predicted to be unstructured, except for the 50 amino acid region that folds into a classical UBA domain. The other insertion between the 5^th^ and 6^th^ blades forms the aforementioned dimerization interface (residues 522–720). Despite a lower local resolution in this region, the EM density map is consistent with the structure prediction from AlphaFold, showing tandem small beta bundles (SBBs), spatially separated but swapped in their primary sequences (Figure S4C). In the context of the native tetramer, four SBBs link across the halves of the ring. Following the RLD, a large helical region forms the core scaffold of UBR5. The eponymous UBR domain, a small motif containing three zinc ions, protrudes from this central region and is also clearly observed in the cryo-EM density map.

As in other HECT-family ubiquitin ligases, the catalytic HECT domain of UBR5 is found at its extreme C-terminus. Several hits from our UBR5 tiling screen (Figure 3A; residues 2,335 and 2,765) cluster in this catalytic domain, highlighting its necessity for RARA degradation. Interestingly, some of these positions are in close proximity to somatic UBR5 mutations observed in mantle cell lymphoma patients.^33^ We mutated the HECT domain of UBR5 and confirmed that it is necessary for ATRA-induced RARA reporter degradation (Figure S5A). In the structure of the UBR5 tetramer, the four HECT domains protrude towards the center of the ring cavity, separated by ~71 Å (within the same side of the ring) or ~91 Å (across opposite sides of the ring). We observe an additional density from an upstream region (residues 2,022–2,067) wedged into a hydrophobic surface on the HECT N-lobe, promoting a predominant L-shaped conformation of the HECT domains (Figures S4D and S4E).^34–38^ This region, which we refer to as the “wedge”, is poised to influence conformational dynamics of the HECT domain over a catalytic cycle and also scores as one of the top hits in our tiling screen (Figure 3A; residue 2,050).

Many ubiquitin ligases harbor UBA domains, which can aid in substrate binding, Ub chain extension or Ub linkage recognition. UBR5 contains a single UBA domain in a highly conserved insertion within the N-terminal RLD that was strongly enriched in our tiling screen (Figure 3A; residue 95). To examine the function of the UBA domain directly, we purified UBR5 with a substitution at the ubiquitin-binding interface (UBR5^V196K^)^31^ or lacking the UBA insertion entirely (UBR5^Δ83–347^). These mutants had severely hindered processivity in ubiquitylation assays and failed to extend a growing ubiquitin chain on a pseudo-ubiquitylated RARA/RXRA substrate (Figure S5B). Our 2D class averages consistently showed a blurry density in the middle of the ring (Figure 3E). This density was notably absent in 2D class averages of UBR5^ΔUBA^, allowing us to assign this to the UBA insertion occupying the center of the ring. Interestingly, this UBA density appears to move within the ring in various 2D-projections and averages out in 3D reconstructions likely due to its conformational variability. We postulate that the multiple UBA domains in a UBR5 tetramer reach toward the center of the ring and provide a binding platform for growing Ub chains to be attacked from the HECT domains, rationalizing the strong Ub branching and extending properties that have been previously described for UBR5.^39,40^

To elucidate the mechanism by which UBR5 recruits NRs, we sought to study these complexes by electron microscopy. Multiple attempts to solve a cryo-EM structure of UBR5 bound to hormone receptors were unsuccessful, possibly due to the dynamic nature, conformationally heterogeneity, and or destruction of these complexes during blotting or freezing processes. In contrast, we observed an additional density corresponding to the size of an RARA/RXRA LBD heterodimer in low-resolution maps of this complex obtained by negative staining (Figures 3F and S5F). In these experiments, we used a dimeric construct of UBR5 lacking both tandem SBBs and HECT domains to eliminate the largest sources of motion in the particle and more easily discern the density corresponding to RARA/RXRA. 3D classifications found the NR density highly dynamic relative to the core of UBR5, partially engaging the helical hinge, RLD propeller, and UBR domains (Figure S5G). UBR5 additionally contains multiple LxxLL motifs in its primary sequence, which could potentially recruit NRs. Although two of these peptide motifs bound RARA/RXRA *in vitro*, these sites are notably buried in inter-helical contacts and would likely exert any effects in an indirect manner (Figures S5C–S5E). Consistent with this, the negative stain envelope additionally demonstrated that UBR5 retains its overall structure following NR binding. Mutagenesis experiments showed deletion of tandem SBB, UBR, or UBA domains individually did not impede agonist-dependent recruitment of RARA, implicating the helical hinge and RLD propeller as an interaction surface (Figure S5H). In the context of the full-length UBR5 tetramer, RARA/RXRA binds proximal to one UBR5 protomer, placing the NR within the ring, adjacent to the HECT domain of its dimerization partner and therefore also in the vicinity of the UBA domains.

### UBR5 is recruited to RARA on chromatin and regulates transcription

Because NCOAs and NRs form complexes on chromatin, we examined whether UBR5 also engages RARA/RXRA on DNA. We assayed UBR5 binding with purified RARA/RXRA in the presence of a fluorescently labeled RA response element (RARE) double-stranded 24-mer DNA fragment and observed that DNA was readily incorporated into a UBR5-RARA/RXRA complex in the presence of ATRA (Figure S6A), suggesting the complex can still associate with DNA through its spatially separated DBD.

**Figure 4.**
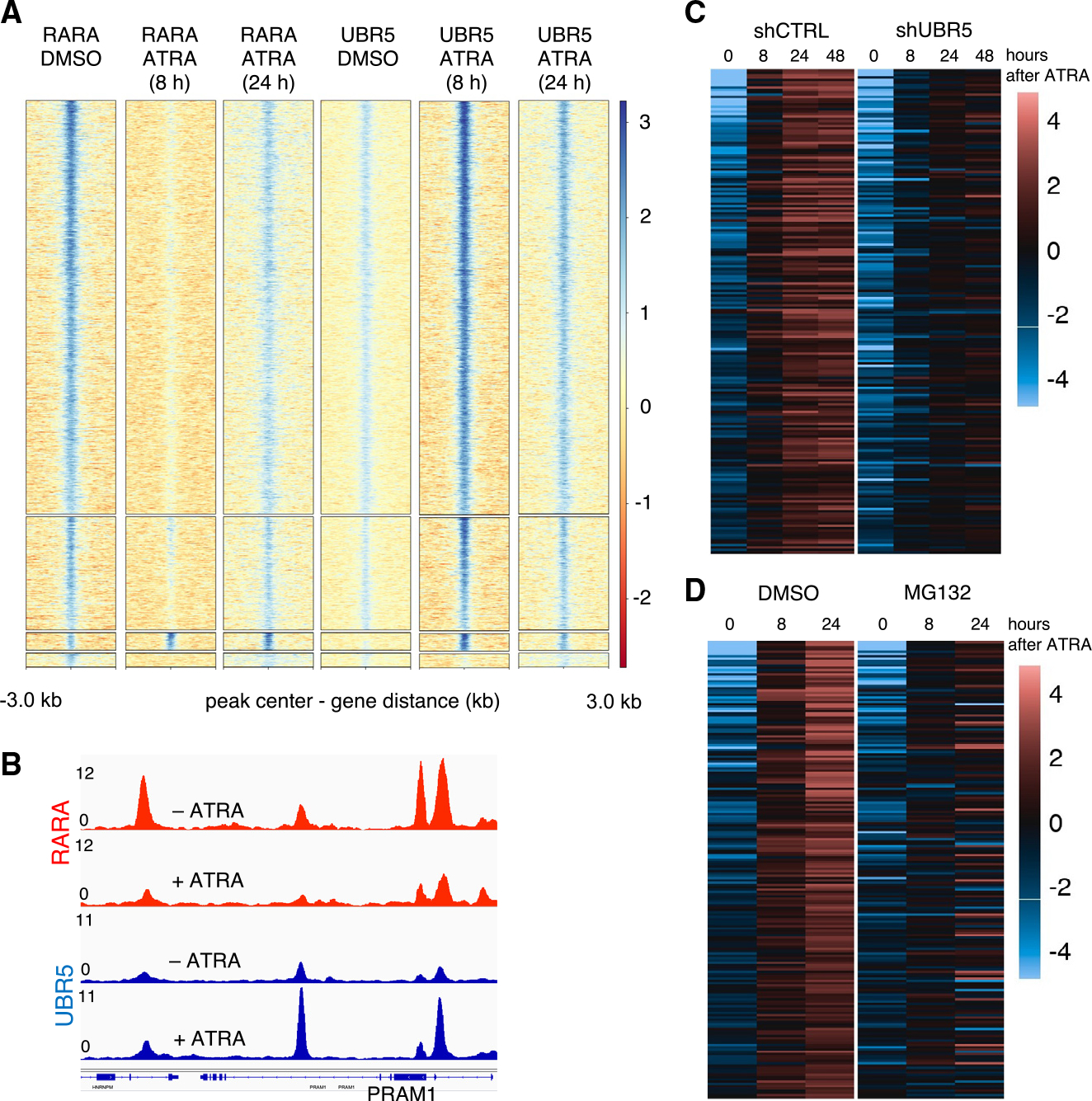
UBR5 binds RARA on chromatin and regulates transcription (A) Tornado plots of chromatin immunoprecipitation sequencing (ChIP-seq) targeting RARA or UBR5 with or without ATRA in NB4 cells, clustered by change following ATRA treatment. Plots are centered around peak center and represent log2 fold change over input (n = 2). (B) ChIP-seq track of RARA (red) and UBR5 (blue) peaks with or without 24 h ATRA treatment. (C) RNA sequencing of NB4 cells transduced with shRNAs against luciferase or UBR5, treated with ATRA for 0, 8, 24, and 48 h. Genes were ordered by increasing expression level changes at 24 h following ATRA treatment in shCTRLs with top 200 genes displayed. Heatmap represents centered log2 scale expression (n = 2). (D) RNA sequencing of NB4 cells treated with MG132 or DMSO for 0, 8, and 24 h following ATRA treatment. Genes were ordered by increasing expression level changes at 24 h following ATRA treatment in shCTRLs with top 200 genes displayed. Heatmap represents centered log2 scale expression (n = 2).

We assessed whether UBR5 co-localizes with RARA on chromatin *in vivo* by chromatin immunoprecipitation sequencing (ChIP-seq) in NB4 cells treated with DMSO or ATRA for 8 and 24 h. Robust peaks for both RARA and UBR5 were identified, demonstrating that that UBR5 is chromatin associated (Figures 4A, 4B, S6B, and S6C). Both RARA and UBR5 were bound primarily at promoters, followed by distal intergenic regions. RARA peaks were most commonly present at baseline and lost following ATRA treatment, consistent with RARA degradation. These loci were also found to bind UBR5, whose binding was strengthened following ATRA treatment, suggesting recruitment and complex formation on chromatin. At fewer loci, both RARA and UBR5 bind only following ATRA treatment, consistent with UBR5 recruitment to RARA sites. A motif search in those peaks showed significant enrichment of four known RARA motifs (Figure S6D). These data are consistent with ligand-induced recruitment of UBR5 to chromatin at RARA-bound sites followed by RARA degradation.

Given the interaction of UBR5 with RARA on chromatin, we examined whether the presence of UBR5 alters transcription of ATRA-regulated genes. RNA sequencing (RNA-seq) was performed on NB4 cells transduced with shRNAs targeting luciferase or UBR5, and treated with ATRA, for 0, 8, 24, or 48 h. Among genes responsive to ATRA in our data (Figure 4C) and previously characterized as ATRA-induced genes^41,42^ (Figure S6E), *UBR5* knockdown strongly decreased transcriptional activation, suggesting a role for UBR5 in ATRA-induced gene expression. To investigate whether these transcriptional changes were caused by degradation^43^ or a different UBR5 function, we performed an analogous RNA-seq experiment with a similar ATRA treatment time course in the presence of MG132. Proteasomal inhibition showed strongly decreased ATRA-induced transcription to that of *UBR5* knockdown (Figure 4D) indicating that the observed transcriptional effects are linked to NR degradation.

### UBR5 regulates a greater subset of NRs through a common degron

**Figure 5.**
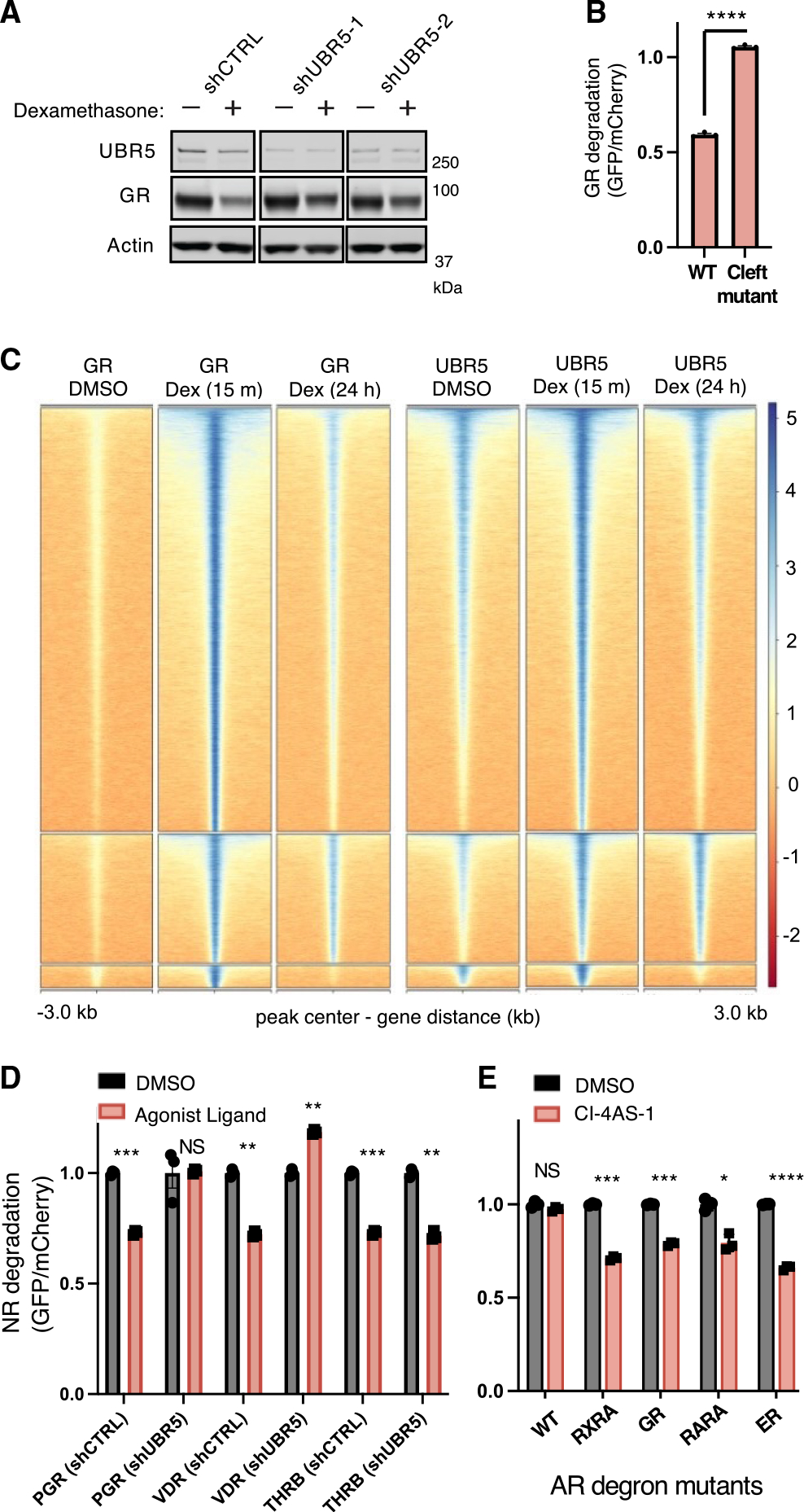
UBR5 regulates a greater subset of NRs through a common degron (A) Western blots of A549 cells transduced with shRNAs against luciferase or UBR5 treated ± dexamethasone. (B) Reporter degradation of WT or hydrophobic cleft mutant GFP-GR reporter (n = 3 two-sided t test). (C) Tornado plots of chromatin immunoprecipitation sequencing (ChIP-seq) targeting GR or UBR5 ± dexamethasone in A549 cells, clustered by change following dexamethasone treatment. Plots are centered around peak center and represent log2 fold change over input. (D) Degradation of indicated fluorescent reporter cell lines transduced with shRNAs against luciferase or UBR5 following indicated ligand treatment (ligands: R5020, calcitriol, and levothyroxine [T3]). (n = 3 two-sided t test). (E) Degradation of fluorescent androgen receptor (AR) reporters with substitutions to residues in the hydrophobic cleft swapped to match those residues found in RXRA, GR, RARA, and ER treated with CI-4AS-1. (n = 3 two-sided t test) (NS, not significant, *p < 0.05, **p < 0.005, ***p < 0.0005, ****p < 0.00005), error bars are SEM.

The UBR5 degron in the H3/H4 hydrophobic cleft is conserved across many NRs. To examine whether other NRs recruit UBR5 to chromatin through similar mechanisms, we examined GR. Dexamethasone promoted binding of UBR5 to GR in cells and *UBR5* shRNAs similarly rescued GR degradation (Figures 5A and S7A–S7D). Mutations of the predicted GR degron, analogous to the RARA degron, impaired dexamethasone-induced GR degradation, as did mutation of the UBR5 HECT domain (Figures 5B and S7E–S7H).

We performed time course co-immunoprecipitation experiments for GR and blotted for NCOA2, GR, and UBR5 at 0, 6, 12, 16, and 24 h following dexamethasone treatment. Similar to our RARA findings, GR sequentially bound NCOA2 and then UBR5 following dexamethasone treatment (Figure S7I). Unlike RARA, which binds constitutively to chromatin, GR is sequestered in the cytoplasm and rapidly translocates to the nucleus following agonist binding.^44,45^ ChIP-seq for GR in A549 cells revealed GR peaks were strikingly gained 15 min after addition of dexamethasone and subsequently decreased following sustained (24 h) dexamethasone treatment, consistent with GR degradation. UBR5 peaks co-localized with GR peaks and strongly increased after 15 min of dexamethasone treatment, suggesting co-recruitment to chromatin (Figure 5C), and its knockdown was also found to alter dexamethasone-regulated gene expression (Figure S7K). Taken together, these data suggest that UBR5 binds and degrades GR in a ligand-dependent manner through a mechanism analogous to the regulation of RARA by UBR5.

Additional NRs that have well-characterized ligand-induced degradation include vitamin D receptor (VDR),^46^ progesterone receptor (PGR),^47^ and thyroid receptor beta (THRB).^7^ To test whether UBR5 regulates these other NRs in a ligand-dependent manner, we created reporter lines and confirmed degradation following treatment with their respective ligands (calcitriol, R5020, and levothyroxine [T3]).^47^ *UBR5* shRNAs rescued degradation for VDR and PGR, and we found that UBR5 binding to VDR *in vitro* was greatly enhanced in the presence of calcitriol, indicating a similar mechanism of UBR5-mediated degradation for these NRs (Figures 5D and S7L–S7N). Notably, T3-induced THRB degradation was not dependent on UBR5 (Figures 5D and S7O), suggesting that other ligases may play a role in the regulation of some NRs or specifically in response to certain ligands.

In contrast to other NRs studied here, androgen receptor (AR) is stabilized by its endogenous ligand testosterone and does not undergo ligand-induced degradation. Instead, it is shuttled out of the nucleus following agonist binding and transcriptional activation.^48–50^ The hydrophobic cleft of AR has several amino acid differences from other NRs, which preferentially accommodate bulkier FxxLF motifs instead of the classic LxxLL.^51,52^ We hypothesized that these sequence differences might also prevent UBR5-mediated degradation and asked whether altering the hydrophobic cleft in AR could confer UBR5 sensitivity following AR agonist treatment. AR reporters with substitutions to the critical set of residues in the hydrophobic cleft (AR V713, V716, V730, and M734) were generated to match those found in RARA, RXRA, or GR. The wild-type AR reporter was not degraded following treatment with the synthetic AR agonist CI-4AS-1. Remarkably, however, the mutant AR reporters were efficiently degraded following treatment with CI-4AS-1 (Figures 5E and S7P). By co-immunoprecipitation, we found increased UBR5 binding at baseline in AR^RARA^ mutants compared with wild-type AR, with strengthened binding in the presence of agonist (Figure S7Q). Taken together, the data underscore the essentiality of the NR hydrophobic cleft for UBR5 binding and NR degradation. Further, our findings demonstrate that the UBR5 degron is transplantable to a previously non-degradable NR, providing the molecular basis for a generalized NR degradation mechanism.

### SERDs engage distinct degradation pathways to degrade ER

**Figure 6.**
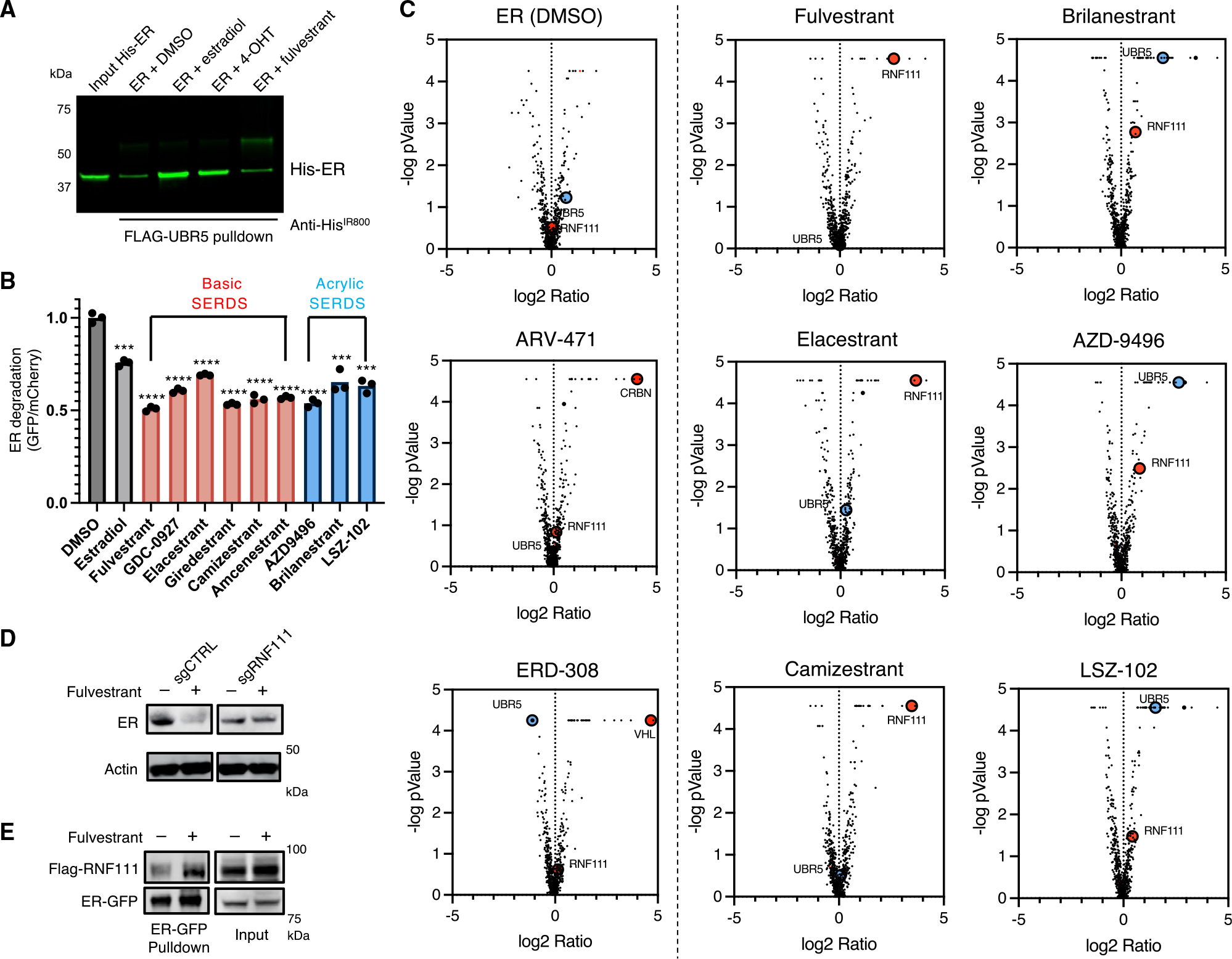
Non-endogenous ligands recruit different E3 ligases to induce ER degradation (A) Western blot showing *in vitro* FLAG-UBR5 pull-down of purified His-tagged ER in the presence of 50 μM indicated ER ligand. (B) ER reporter degradation following 1 μM of specified SERD treatment. (C) Volcano plots of targeted CRISPR screens highlighting enrichment of UBR5 or RNF111 following SERD treatment of ER fluorescent reporter K562 cell lines. UBR5 is highlighted in blue, RNF111, CRBN, VHL are highlighted in red. (D) Western blots of T47D cells transduced with sgRNAs against RNF111 treated with or without fulvestrant. (E) Co-immunoprecipitations of 293Ts co-transfected with FLAG-RNF111 and ER-GFP treated ± fulvestrant and proteasome inhibitor (MG132) and blotted with antibodies specific for FLAG or GFP as indicated. (NS, not significant, *p < 0.05, **p < 0.005, ***p < 0.0005, ****p < 0.00005, error bars represent SEM.)

SERDs are a class of molecules targeting the ER for the treatment of breast cancer. Two SERDs, fulvestrant^17^ and elacestrant,^53^ lead to ER degradation and have been approved by the FDA. Alkyl tail containing SERDs, including fulvestrant, destabilize helix 12 and block the ER hydrophobic cleft, preventing coactivation.^54^ Given that UBR5 is similarly recruited to the NR hydrophobic cleft, we hypothesized that fulvestrant and other SERDs may employ a different mechanism for ER degradation. In a direct binding assay with recombinant proteins, the UBR5-ER interaction was induced by estradiol but not fulvestrant, suggesting that fulvestrant-driven ER degradation employs a different E3 ligase (Figure 6A).

To characterize the UPS pathway components involved in SERD-induced ER degradation, we performed CRISPR screens with our ER reporter in the presence multiple SERDs: fulvestrant, GDC-0927, elacestrant, giredestrant, camizestrant, amcenestrant, AZD-9496, brilanestrant, and LSZ-102. All SERDs and the ER-targeting proteolysis targeting chimeric (PROTAC) molecules ARV-471 and ERD-308 induced ER reporter degradation (Figure 6B). Genetic screens revealed that SERDs with acrylic acid side chains (AZD-9496, brilanestrant, and LSZ-102) required UBR5 for degradation, whereas fulvestrant and SERDs with basic amino moieties (GDC-0927, elacestrant, giredestrant, amcenestrant, and camizestrant) employed a different E3 ligase, RNF111 (Figures 6C and S8A). As controls, the PROTACs ARV-471 and ERD-308 were found to utilize Cereblon (CRBN) and von Hippel-Lindau (VHL), respectively (Figures 6C and S8B). To validate these results, we demonstrated that knockdown of *RNF111* or *UBR5* rescued ER degradation by basic amino or acrylic acid SERDs, respectively, in reporter assays and of endogenously expressed ER in breast cancer cells (Figures 6D and S8C–S8F). We found that fulvestrant induces association of RNF111 with ER *in vivo* by BRET (Figure S8G) and co-immunoprecipitation (Figure 6E). Similarly, brilanestrant increased ER and UBR5 interactions by co-immunoprecipitation (Figure S8H). Taken together, these findings indicate that different classes of SERDs engage distinct ubiquitin ligases and degradation pathways to target ER.

## DISCUSSION

In this study, we identify UBR5 as a general, ligand-dependent regulator of multiple NRs. Agonist binding to NRs induces conformational changes in their LBDs, leading to the exposure of a hydrophobic cleft that recruits both NCOAs and UBR5. UBR5 ultimately competes with and displaces NCOAs leading to NR degradation. Based on the sequence conservation of the NR binding site, we propose that UBR5 acts as a ligand-activated E3 ligase for a broader number of NRs beyond the seven examples shown herein. We additionally show the UBR5/NR degron can be transplanted to the AR, a non-agonist-degraded NR, to confer susceptibility to UBR5 degradation. The interactions between UBR5 and NRs occur on chromatin, where UBR5 acts as a regulator of transcription in addition to NR protein stability.

SERDs are highly effective therapies for breast cancer. One of the best understood SERDs, fulvestrant, drastically remodels the hydrophobic cleft^16,17^ in a manner incompatible with UBR5 or NCOA binding. We found that different SERDs engage separate degradation pathways represented by UBR5 and RNF111. Strikingly, this correlates with clinical utility, hallmarked by the wide use of fulvestrant and recent approval of elacestrant,^53^ both of which employ RNF111, in contrast to UBR5 SERDs (Table S2), none of which have been approved for clinical use. The heterogeneity of ER transcriptional profiles following SERD treatment has previously been characterized; fulvestrant was shown to be the most antagonistic of tested SERDs, whereas SERDs using UBR5 led to at least partial ER agonism.^17,55^ We therefore speculate that, in accordance with our findings, UBR5 SERDs lead to ER activation by first allowing for NCOA binding. SERDs that do not allow UBR5 binding destabilize helix 12, preventing both NCOA and UBR5 accessibility,^54^ leading to the recruitment of a different ligase and ER antagonism. Because we have shown UBR5’s engagement to be generalizable, development of novel NR ligands could selectively recruit UBR5 or other ligases to favor agonism or antagonism associated degradation pathways.

UBR5 supports binding of diverse substrates, such as NRs,^56,57^ basic-helix-loop-helix (bHLH) proteins Myc/Max,^58,59^ and components of the mitotic checkpoint complex,^60^ through multiple, likely distinct, binding sites without the requirement for additional substrate receptors. In an accompanying manuscript, UBR5 was shown to mediate the degradation of a broader number of transcription and chromatin associated factors including MYC, MCRS1, STP4, SPT5, and CDC20.^61^ UBR5 recognizes these substrates through degrons concealed by binding partners or chromatin, analogous to the shielding of the NR degrons by helix 12 and exposure of the degron following ligand binding. Many of these factors are found at gene regulatory sites overlapping with that of hormone receptors. The size of the UBR5 tetramer in conjunction with its flexible UBA and HECT modules in the ring could therefore allow UBR5 to impact the stability of adjacent proteins on chromatin, as supported by the UBR5 structures with RARA/RXRA (Figure 3F) and of UBR5 bound to MCRS1.^61^ Integration of findings from these two manuscripts suggests that UBR5 is recruited to chromatin via hormones to regulate NRs. UBR5 may then engage other adjacent transcription and chromatin associated factors with UBR5 degrons in order to remodel promoters and transcriptional complexes more broadly. UBR5 can thereby integrate multiple hormonal and stress-related signals as a regulatory hub on chromatin.

The role of UBR5 in regulating many NRs, especially following the administration of therapeutics such as ATRA and corticosteroids, highlights its clinical and physiologic importance. The broad set of targets engaged by UBR5 provides an elegant model for cellular responses to many hormones and an integration of transcriptional activity from multiple signals. A single, highly processive ligase targetable to loci by small molecules permits rapid turnover of transcriptionally active NR complexes, priming cells to respond quickly to new pulses of hormones. Our findings demonstrate a pivotal role of protein degradation regulating the transcriptional response of NRs and the potential for the rational design of small molecules that modulate ligand-dependent UBR5 recruitment, degradation, and consequently NR-mediated transcription.

### Limitations of the study

Our joint studies demonstrate that UBR5 functions on chromatin; however, a limitation is that the exact nature of UBR5 as a transcriptional hub must be studied further. Although we show the mechanism by which UBR5 is recruited to chromatin in response to nuclear hormones and demonstrate its interactions between it and specific substrates, such as nuclear receptors and MCRS1, future high-resolution structures of UBR5 bound to NRs, coupled with further studies of UBR5/substrate chromatin localization, will permit further dissection of our proposed model of the regulation of multiple transcription factors by UBR5 at a single locus. Finally, although we have demonstrated direct competition between NCOAs and UBR5 for NR binding, the exact NR residues UBR5 engages is unclear because we are currently limited by the resolution of our NR-bound UBR5 structure. This leads to an intriguing line of investigation, where differential binding of NCOAs and UBR5 may be tunable by small molecules. These studies should allow us to better interrogate the role of UBR5 in integrating multiple signals to govern transcription, with the ability to modulate NR signaling by rationally controlling receptor degradation.

## STAR★METHODS

### RESOURCE AVAILABILITY

#### Lead contact

Further information and requests for resources and reagents should be directed to and will be fulfilled by the lead contact, Benjamin L. Ebert (benjamin_ebert@dfci.harvard.edu).

#### Materials availability

All reporter and expression plasmids used in this study will be made available on request. This study did not generate new unique reagents.

#### Data and code availability

- The EM density maps have been deposited at the Electron Microscopy Data Bank database with accession code EMD-17539 (UBR5 dimer), EMD-17540 (UBR5 tetramer), and EMD-17542 (negative stain map of UBR5 dimer with RARA/RXRA). Corresponding atomic models were deposited at the RCSB Protein Data Bank database with accession codes 8P82 (UBR5 dimer) and 8P83 (UBR5 tetramer). Proteomics data sets are deposited in PRIDE with the accession numbers PXD040953, PXD041749. ChIP and RNA sequencing data are deposited in GEO with the accession number: GSE213795 (GSE213742 – ChIPseq, GSE213793 – RNAseq). All data are publicly available as of the date of publication. Accession numbers and DOI are listed in the key resources table.
- This paper does not report original code.
- Any additional information required to reanalyze the data reported in this paper is available from the lead contact upon request.

### EXPERIMENTAL MODEL AND STUDY PARTICIPANT DETAILS

Recombinant UBR5 (and mutants thereof) for biochemical and structural studies were produced in HEK293F cells via transient expression using the Expi293 Expression System Kit (Thermo Fisher) following the manufacturer’s protocol. Recombinant nuclear receptor proteins (RARA, RXRA, GR, ER, VDR as LBD or DBD-LBD constructs) were produced in E. coli BL21-CodonPlus(DE3)-RIL cells grown in LB broth media. Recombinant NCOA1 proteins were produced in Trichoplusia ni and Spodoptera frugiperda insect cells cultured at 27°C in SF4 Baculo Express Media (BioConcept). Functional genomic screens were performed in U937-Cas9 cells provided by the Genetic Perturbation Platform, Broad Institute. Chromatin-immunopreciptiation sequencing (ChIPseq) and RNA sequencing experiments were performed in NB4 cells, provided by the Genetic Perturbation Platform, Broad Institute. A549 cells were purchased directly from ATCC. Cells were authenticated by the Molecular Diagnostics Laboratory (MDL) at Dana-Farber Cancer Institute via high resolution small tandem repeat (STR) profiling compliant with American National Standards Institute (ASN-0002) recommendations. U937 (male), NB4 (female), A549 (male) cell lines were grown in RPMI with 10% FBS and 1% glutamine and penicillin-streptomycin at 37°C and 5% CO2.

### METHOD DETAILS

#### Functional genomics screens

The UPS and tiling CRISPR functional genomics screen was performed following previously described methods.^22^ Briefly, the UPS CRISPR library targets 713 E1, E2, E3 ubiquitin ligases, and deubiquitinases and includes controls genes with a total of 2852 guide RNAs, cloned into the pXPR003 backbone. The UBR5 Tiling screen was created by CRISPR-SURF.^29^ Viruses were produced in a T-175 format and ten percent by volume of the library virus was added to reporter cell lines in triplicates for transduction. Transduced cells were allowed to recover and expand for nine days, then treated with indicated ligand. Top and bottom 5% of cells by GFP signal were sorted for a total of at least 100,000 cells per replicate. Samples were processed as previously described.^22^

Reporter cell lines were treated with ATRA or 9-*cis* RA, and then sorted into four populations by eGFP/mCherry fluorescence ratios (Gates A-D), corresponding to the bottom and top 5–10% respectively (Figure S1D).^22,23^ Sorted cells were pelleted, lysed, and processed according to previous studies.^22^ Briefly sgRNAs were amplified in two PCRs via Titanium Taq (Takara Bio 639210), Titanium Taq Buffer and 800uM dNTP mix and eight staggered forward primers. The first PCR also contained 200 nM SBS3-Stagger-pXPR003 forward primer and 200 nM SBS12-pXPR003 reverse primer (cycles: 5 minutes at 94°C, 15 × (30 sec at 94°C, 15 sec at 58°C, 30 sec at 72°C), 2 minutes at 72°C). Illumina adapters and barcodes were added in the second PCR (200 nM P5-SBS3 forward primer, 200 nM P7-barcode-SBS12 reverse primer). Samples were pooled and purified by agarose gel-electrophoresis (Qiagen) and NaOAc/isopropanol precipitation. Amplified guides were quantified by next-generation sequencing (Genomics Platform, Broad Institute), and analyzed for enrichment in Gate A over Gate D, representing degradation rescue.

#### Mammalian cell culture

The human HEK293T, U937-Cas9 and NB4-Cas9 cells were provided by the Genetic Perturbation Platform, Broad Institute. The A549 cells were purchased from ATCC. HEK293Ts were cultured in Dulbecco’s modified Eagle’s medium (DMEM (Gibco)), and all other cell lines were cultured in RPMI (Gibco) with 10% fetal bovine serum (Invitrogen), and 5% glutamine and penicillin-streptomycin (Invitrogen) at 37°C and 5% CO2.

#### Reporter vectors

The following reporters were used in this study: SFFV.BsmBICloneSite-10aaFlexibleLinker-eGFP.IRES.mCherry.cppt.EF1a.PuroR, SFFV.eGFP.BsmBICloneSite-10aaFlexibleLinker-.IRES.mCherry.cppt.EF1a.PuroR for NR reporter vectors, sgRNA.SFFV.Puro, sgRNA.SFFV.tBFP for gRNAs, and pLKO.1 (Broad Institute, Addgene 10878) for shRNAs.

#### Cloning, lentiviral packaging, and reporter cell line generation

Constructs were generated by BsmBI (New England Biolabs) restriction digest of both vectors and inserts, and ligated with T4 DNA Ligase (New England Biolabs). Constructs were transformed into Stbl3 *Escherichia coli* and sequences were confirmed by Sanger sequencing. Lentiviruses for reporters, sgRNAs, shRNAs, were packaged as previously described.^22^ Briefly, 500,000 HEK293T cells were seeded in 2ml of DMEM media supplemented with 10% FBS and pencillin-streptomycin-glutamine. A packaging mix included 1500 ng of psPAX2, 150ng of pVSV-G, and 1500 ng of vector was prepared in 37.5 μl of OptiMem (Thermo Fischer). This mix was combined with 9ul of TransIT-LT1 (Mirus) and 15 μl of OptiMem and incubated for 30 minutes at room temperature and then applied drop-wise to cells. Cells were allowed to incubate for 48 hours. Lentivirus was collected by 0.4 μm filters.

#### Degradation assays

Reporter cell lines were seeded in triplicate at a density of 1 million cells/ml in 100 μl per well in a 96 well plate and measured once. Ligand was added by Digital Dispenser (Tecan), and incubated at 37°C for the appropriate time before reading by flow cytometry. Cells were analyzed for GFP vs mCherry expression. GFP expression was normalized to mCherry signal and ligand treatments were compared to DMSO controls.

#### Immunoblots

Cells (1–5 million) were washed with PBS and lysed in Pierce IP Lysis Buffer (ThermoFisher, 87787) with 1x Halt Cocktail protease (ThermoFisher, 87786) for 30 minutes on ice, and centrifuged for 15 minutes at maximum speed to remove the insoluble fraction, or processed via NE-PER Nuclear and Cytoplasmic Extraction Kit (ThermoFisher, 78833) according to manufacturer’s instructions. Protein concentration was quantified via Pierce BCA Protein Assay Kit (ThermoFisher, 23225), and equal amounts of lysates were run on either SDS-PAGE 3–8% Tris-Acetate or 4–12% Bis-Tris Protein Gels (NuPAGE, ThermoFisher), then transferred to a PVDF membrane (Biorad, Wet Transfer System). Membranes were blocked for 30 minutes to 1 hour at room temperature in Odyssey Blocking Buffer/PBS (LI-COR Biosciences), and incubated with primary antibodies at 4 °C overnight. Stained membranes were washed three times in Tris-buffered saline with Tween 20 (TBS-T) and incubated for 1 hour at room temperature with secondary IRDye-conjugated antibodies (LI-COR Biosciences). Membranes were washed three times in TBS-T before imaging (Odyssey Imaging System, LI-COR Biosciences).

#### Co-immunoprecipitation assay

Reporter cells (up to 100 million) were treated with ligand (ATRA, dexamethasone) and allowed to incubate for different time points. Cells were washed with PBS with ligand added and lysed in Pierce IP Lysis Buffer (ThermoFisher, 87787) with 1x Halt Cocktail protease (ThermoFisher, 87786) in the presence of ligand for 30 minutes on ice, and centrifuged for 15 minutes at maximum speed to remove the insoluble fraction. Lysates were incubated with GFP-Trap Magnetic Agarose beads (ChromoTek, GTMA-20) overnight at 4°C on a rotator. Beads were magnetically removed and washed three times with Pierce IP Lysis Buffer with ligand added before boiling in 1x NuPAGE LDS Sample Buffer (ThermoFischer, NP0007). Blotting was done as described above. Experiments were repeated and representative images are shown.

#### Bioluminescence resonance energy transfer analyses

Bioluminescence resonance energy transfer (BRET) experiments were done using the NanoBRET PPI kit (Promega, N1821) according to manufacturer’s instructions.

#### Chromatin immunoprecipitation sequencing

Cell lines were expanded (up to 50 million), treated with ligand, and incubated at 37 °C. Cells were pelleted by centrifugation for 5 minutes, washed twice with cold PBS, and resuspended, in 5 ml of room temperature PBS. 20 μl of 0.5M Disuccinimidyl glutarate (DSG) (ThermoFisher), was added and incubated for 30 minutes at room temperature. Cells were pelleted by centrifugation and resuspended in 8.325 ml of PBS and 8.325 ml of 2% formaldehyde and incubated for 30 minutes at room temperature. Cross-linking was quenched by the addition of 1665 μl of 1 M Tris-HCl pH 8.0 and 832.5 μl of 2.5 M glycine. Cells were pelleted and washed twice with PBS. Cross-linked cells were resuspended in LB1 Buffer (50mM HEPES-KOH, pH7.5, 140mM NaCl, 1mM EDTA, 10% Glycerol, 0.5% NP-40, 0.25% Triton X-100 in distilled water) and rotated for 10 minutes at 4C. Cells were pelleted and resuspended in LB2 (10mM Tris-HCl, pH 8.0, 200mM NaCl, 1mM EDTA, 0.5mM EGTA in distilled water) and rotated for 10 minutes at 4C. Cells were pelleted and resuspended in LB3 (10mM Tris-HCL, pH 8.0, 100mM NaCl, 1mM EDTA, 0.5mM EGTA, 0.1% Na-deoxycholate, 0.5% Na-Lauroylsarcosine, 1% Triton X-100, in distilled water) sonicated (Covaris, E220). Samples were transferred into DNA LoBind tubes (Eppendorf) and clarified by centrifugation. Antibody-bead conjugates were made with either Protein-A or Protein-G Dynabeads (ThermoFisher). Beads were washed twice with ChIP dilution buffer (0.01% SDS, 1.1% Triton X-100, 1.2 mM EDTA, 16.7 mM Tris-HCl pH 8.1, 167 mM NaCl in distilled water) and resuspended with the appropriate amount of beads (according to manufacturer’s instructions), and rotated overnight at 4C. Antibody-bead complexes were applied to lysates and rotated overnight at 4C. Beads were removed via magnet and washed six times with RIPA buffer (50mM Hepes-KOH, pH 7.5, 500mM LiCl, 1mM EDTA, 0.7% Na-deoxycholate, 1% NP-40 in distilled water), twice with Buffer 500 (0.5g deoxycholic acid, 1mM EDTA, 5mM Tris-HCl, pH 8.1, 1% Triton X-100, and 0.02% NaN3 in distilled water), and twice with LiCl Buffer (2.5 g deoxycholic acid, 1 mM EDTA, 250 mM LiCl, 0.5% NP-40, 10 mM Tris-HCl pH 8.1, 0.02% NaN_3_ in distilled water). Beads were washed briefly with TE buffer and DNA was eluted in ChIP elution buffer (50mM Tris-HCl, pH 8.0, 10mM EDTA, 1% SDS in distilled water) and de-crosslinked overnight at 65C. DNA was purified by column purification (DNA Clean and Concentration, Zymo Research). Library preparation was done according to manufacturer’s instructions (ThruPlex DNA-Seq Kit, Takara). Fragment length and concentrations were assessed using TapeStation (Agilent) and Qubit (ThermoFisher), and libraries were sequenced on an Illumina NextSeq 550 sequencer.

#### RNA sequencing

Cell lines were expanded, treated with ligand, and incubated at 37C. Cells were pelleted by centrifugation for 5 minutes, washed with cold PBS, and RNA was isolated by RNeasy kit (Qiagen) per manufacturer’s instructions. RNA was sent to Novogene for library preparation and sequencing, or prepared for sequencing (NEBNext, New England Biolabs) and sequenced on an Illumina NextSeq 550 sequencer.

#### Purification of recombinant NRs

RARA, RXRA, GR, VDR, ER (LBD or DBD-LBD constructs) and mutants thereof were recombinantly expressed in E. coli BL21-CodonPlus(DE3)-RIL cells (Agilent) as His_6_–Smt3 fusion proteins. Cells were lysed by sonication in lysis buffer containing 50 mM Tris pH 8.0, 500 mM NaCl, 5% Glycerol, 0.5 mM TCEP, 50 mM Imidazole and 1× protease inhibitor cocktail (Sigma). The lysate was cleared by ultracentrifugation and subjected to a HisTrap FF column (Cytiva), followed by washing with 50 mM Tris (pH=8.0), 500 mM NaCl, 5% Glycerol, 0.5 mM TCEP and 50 mM Imidazole and eluted in 50 mM Tris (pH=8.0), 500 mM NaCl, 5% Glycerol, 0.5 mM TCEP and 400 mM Imidazole. In all cases, size-exclusion chromatography was performed as a final step in 25 mM HEPES (pH 7.4), 150 mM NaCl 0.5 mM TCEP with 5% Glycerol added as a cryopreservative.

#### UBR5 expression and purification

Full-length human UBR5 (or mutants and truncations thereof) with an N-terminal FLAG tag were cloned into a pDEST-CMV vector (Thermo Fisher) for transient expression in mammalian cells. UBR5 was expressed in HEK293F cells in a suspension culture of 0.5 L scale following the protocol from Expi293 Expression System (Thermo Fisher). Cells were harvested three days post-transfection by centrifugation and lysed by gentle sonication in 60 mL “lysis buffer” (25 mM HEPES pH 7.6, 150 mM KCl, 5 mM MgCl_2_, 0.5 mM CaCl_2_, 5% Glycerol, 0.1% TRITON X-100) supplemented with protease inhibitor cocktail (Sigma). The lysate was cleared by ultra-centrifugation at 35 K rpm for 45 minutes and the supernatant filtered through a 1.2 μM syringe filter. The filtered lysate was incubated with 5 mL of FLAG M2 affinity gel (Sigma) for 2 hours at 4°C with gentle rotation, after which the affinity gel was collected in a glass column for further washing. Following initial washes with lysis buffer, the column was washed with “wash buffer” (25 mM HEPES pH 7.6, 5% Glycerol, 0.05% TWEEN-20) supplemented with 700 mM KCl. Finally the column was washed in wash buffer supplemented with 300 mM KCl, and eluted with wash buffer supplemented with 300 mM KCl and 0.3 mg/mL 3xFLAG peptide.

FLAG elution fractions containing UBR5 protein were applied directly to a MONO-Q 5/50 GL column (Cytiva) equilibrated in wash buffer + 300 mM KCl + 0.5 mM TCEP. The protein was eluted using a linear gradient from 300 mM to 800 mM KCl, with UBR5 eluting near a salt concentration of 500 mM KCl. Fractions containing UBR5 were collected, concentrated and injected onto a Superose 6 Increase 10/300 (Cytiva) column in storage buffer (25 mM HEPES pH 7.6, 150 mM KCl, 0.5 mM TCEP and 5% Glycerol). The final yield of UBR5 was typically 2–4 mg of protein from a 0.5 L mammalian cell culture volume.

#### NR/UBR5 binding assays *in vitro* with purified proteins

12 μL of FLAG M2 slurry were equilibrated with binding buffer (25 mM HEPES pH 7.4, 125 mM NaCl, 0.05% TWEEN-20). The resin was pelleted and excess buffer removed. 60 μL of fresh binding buffer was re-added, followed by addition of 15 pmol (2.5 μL ofa6 μM solution) of purified FLAG-UBR5 protein and incubated on a rotating wheel at 4°C for 60–90 minutes. The resin was pelleted, excess buffer removed, and 150 μL fresh binding buffer (supplemented with 50 μM ligand of interest or DMSO) added, followed by 150 pmol protein (binding partner protein of interest) and incubated for another 60 minutes. The resin was pelleted, excess buffer removed and washed with 400 μL of binding buffer (supplemented with ligand of interest or DMSO), and this process was repeated three times. The resin was pelleted, excess buffer removed and 45 μL of binding buffer supplemented with 1 mg/mL 3xFLAG peptide was added to elute proteins over 30 minutes on a rotating wheel at room temperature. The resin was pelleted, and 40 μL of the supernatant was collected and mixed with 12 μL of 5xSDS gel loading buffer. 6 μL of these samples were applied to SDS-PAGE on a 4–20% gradient gel (Biorad). The gel was transferred to a nitrocellulose membrane using the Trans-Blot Turbo Transfer System (Biorad). Following blocking with 5% skim milk solution in TBST, primary antibodies targeting the NR proteins were added in TBST and incubated overnight at 4 °C. The following day, membranes were washed thrice with TBST buffer and incubated for 45 minutes with fluorescent secondary antibody (Invitrogen A11357), followed by three more washes with TBST. Imaging was performed on a Licor Odyssey system, scanned at 800 nm.

#### Fluorescence polarization experiments

Fluorescein-labelled NCOA1 peptide residues 687–698 (Flc-ARHKILHRLLQEGS) was used as a fluorescent tracer as described previously.^71^ Increasing amounts of RARA/RXRA heterodimer (0.1–26 μM final concentration) were mixed with tracer (10 nM final concentration) in a 384-well microplate (784076, Greiner) at room temperature. The interaction was measured in a final buffer containing 20 mM HEPES pH 7.4, 110 mM NaCl, 500 μM TCEP, 1% Glycerol, 0.1% (v/v) pluronic acid with or without 100 μM all-*trans*-retinoic acid. A PHERAstar FS microplate reader (BMG Labtech) equipped with a fluorescence polarization filter unit was used to determine changes in fluorescence polarization. The polarization units were converted to fraction bound, defined as (mP_free_ – mP) / (mP_free_ – mP_bound_), as described previously.^72^ The fraction bound was plotted versus protein concentration and fitted assuming a 1:1 binding model to determine the dissociation constant (*K*_d_) using Prism 9 (GraphPad). For the competitive reverse titration assays, RARA/RXRA bound to the fluorescent oligo tracer was back-titrated with unlabeled UBR5 peptides (see Figure S5D or key resources table for exact sequences) or the same NCOA1 peptide lacking a fluorescein label. These counter-titration experiments were carried out by mixing tracer (10 nM) and RARA/RXRA (0.5 μM), and titrating increasing concentration of the unlabeled competitor peptides (0–85 μM). The fraction bound was plotted versus competitor concentration and the data were fitted with a non-linear regression curve (variable slope) to obtain K_app_ values in Prism 9 (GraphPad). All measurements were performed in duplicates. Experiments with the glucocorticoid receptor were performed identically but using a fluorescein-labelled NCOA2 peptide (residues 741–753) as a fluorescent tracer and dexamethasone included as an agonist ligand.

#### Cryo-EM

UBR5 proteins (with or without RARA/RXRA and corresponding agonist ligands at 50 μM) at ~2 mg/mL were applied to 10–40% glycerol gradients composed of 20 mM HEPES pH 7.6, 150 mM NaCl, 0.5 mM TCEP and 0–0.1% glutaraldehyde according to the “GraFix” method.^73^ Gradients were ultracentrifuged at 30 K rpm for 14h (for tetrameric UBR5) or 38 K rpm for 16h (for dimeric UBR5) in a Sw-60 swinging bucket rotor. Gradients were harvested top-down by piston fractionation (BioComp) and peak fractions monitored by absorbance traces and/or SDS-PAGE. Peak fractions were pooled and buffer exchanged using Zeba-spin desalting columns (Thermo Fisher) into buffer without glycerol (20 mM HEPES pH 7.6, 150 mM NaCl, 0.5 mM TCEP, 0.01% fluorinated octyl maltoside). Finally, samples were concentrated to ~30 μL using Amicon centrifugal filter concentrators (Merck-Millipore).

3μL of sample were applied to freshly glow-disharged UltrAuFoil 1.2/1.3 300-mesh grids (Quantifoil) or 1.2/1.3 Cu 200-mesh grids (Quantifoil) with or without a 2nm layer of amorphous carbon. A Vitrobot Mark IV (FEI) was used for plunge freezing into liquid ethane following a 3s blot time at 95% humidity and 4 °C.

All data were collected on a Cs-corrected (CEOS GmbH, Heidelberg, Germany) Titan Krios electron microscope (Thermo Fisher) operated at 300 kV. Data collection were performed automatically using the EPU software package (Thermo Fisher) at a nominal magnification of 59,000x, corresponding to a pixel size of 1.1 Å. Movies recorded with a Falcon 4 direct electron detector (Thermo Fisher) in 50 frames with a total dose of 50 e^−^/Å^2^, except for data collected at a stage tilt of 30˚,°which had a total dose of 55 e^−^/Å^2^. Targeted defocus values ranged from −1.0 to −2.2 μm. Real-time evaluation was performed with CryoFLARE,^62^ and micrographs below an EPA limit of 6 Å were retained for further processing. All additional processing steps were performed in cryoSPARCv3,^63^ with additional processing pipeline details found in Figure S3. Reported resolution values are based on the gold-standard Fourier shell correlation (FSC) curve at 0.143 criterion.^74^ High-resolution noise substitution was used for correcting the effects of soft masking for the related FSC curve. Local resolutions were estimated using cryoSPARC. Cryo-EM maps were sharpened by local density sharpening with LocScale^64^ for visualization.

#### Cryo-EM model building

The structure for full-length monomeric UBR5 was first predicted with AlphaFoldv2^65^ and docked into the UBR5 dimer cryoEM map using ChimeraX fit-in-map.^68^ After restrained flexible-fitting with ISOLDE,^66^ local corrections of side chains and segments were done with Coot^70^ and ISOLDE. Using an intermediate model as template, the dimer of the helical bundle domain was predicted with AlphaFold-multimer^75^; using an in-house interface (https://github.com/fmi-basel/GUIFold) and then used as an additional guide. The model was iteratively refined by local rebuilding in Coot/ISOLDE and minimization with Rosetta FastRelax^69^ in combination with density scoring using an in-house pipeline (https://github.com/fmi-basel/RosEM) followed by coordinate-constrained minimization with Phenix real-space refine.^67^ B-factors were fitted at a final stage with Rosetta. Unobserved side-chains were marked with zero occupancy.

To build a model for the complete tetrameric UBR5 structure, the model from the higher-resolution (dimer) structure was docked with ChimeraX fit-in-map. The tandem domain dimer was predicted separately with AlphaFold-multimer, and then flexibly fitted in ISOLDE using self-restraints. A single tandem domain subunit was merged with the remaining part of an adjacent UBR5 subunit and used as input for symmetric refinement with Rosetta FastRelax in torsional and cartesian space (including density scoring as above). The remaining steps were as described above. Side-chains were truncated to Cβ. Validation was carried out with Phenix,^76^ Molprobity,^77^ and EMRinger.^78^ Figures were prepared with PyMOL (Schrödinger, LLC.) and ChimeraX.

#### Negative staining

Complexes of UBR5^ΔtandemΔHECT^, with or without RARA/RXRA^LBD^ and retinoic acid, were applied to GraFix Glycerol gradients and prepared as described above. 4μL of samples at 0.03 mg/mL were applied to Formvar/Carbon 300 mesh copper grids (#01753-F, Ted Pella) glow discharged with a Pelco EasyGlow (15 mA negative current, 45 s) (Ted Pella) for 30 seconds. Side blotting was performed to remove excess liquid, followed by 2 successive washes of buffer and 3 successive applications of 2% uranyl acetate for 20 seconds, with side blotting to remove excess liquid. Once dried, data were acquired with a Tecnai Spirit (FEI) transmission electron microscope operated at 120 keV. 300–400 images were recorded with an Eagle camera (FEI) at a nominal magnification of 49,000× resulting in a pixel size of 2.1 Å. Images were recorded by varying the defocus between −1.0 and −2.5 μm. Data were subsequently processed in cryoSPARCv4, using the suggested processing pipeline for negative stain data. Following homogeneous refinement for a consensus map, 3D classification was performed in cryoSPARCv4 to reveal the conformational variability presented in Figure S5.

#### Sample preparation LFQ quantitative mass spectrometry

NB4 cells were treated with DMSO or 0.1, 1, or 10mM ATRA for 24 h. Cells were harvested by centrifugation and washed with phosphate buffered saline (PBS) before snap freezing in liquid nitrogen.

Cells were lysed by addition of lysis buffer (8 M Urea, 50 mM NaCl, 50 mM 4-(2-hydroxyethyl)-1-piperazineethanesulfonic acid (EPPS) pH 8.5, Protease and Phosphatase inhibitors) and homogenization by bead beating (BioSpec) for three repeats of 30 seconds at 2400. Bradford assay was used to determine the final protein concentration in the clarified cell lysate. 50 mg of protein for each sample was reduced, alkylated and precipitated using methanol/chloroform as previously described^79^ and the resulting washed precipitated protein was allowed to air dry. Precipitated protein was resuspended in 4 M Urea, 50 mM HEPES pH 7.4, followed by dilution to 1 M urea with the addition of 200 mM EPPS, pH 8. Proteins were first digested with LysC (1:50; enzyme:-protein) for 12 h at RT. The LysC digestion was diluted to 0.5 M Urea with 200 mM EPPS pH 8 followed by digestion with trypsin (1:50; enzyme:-protein) for 6 h at 37 °C. Sample digests were acidified with formic acid to a pH of 2–3 prior to desalting using C18 solid phase extraction plates (SOLA, Thermo Fisher Scientific). Desalted peptides were dried in a vacuum-centrifuged and reconstituted in 0.1% formic acid for LC-MS analysis.

Data were collected using a TimsTOF Pro2 (Bruker Daltonics, Bremen, Germany) coupled to a nanoElute LC pump (Bruker Daltonics, Bremen, Germany) via a CaptiveSpray nano-electrospray source. Peptides were separated on a reversed-phase C_18_ column (25 cm × 75 μm ID, 1.6 μM, IonOpticks, Australia) containing an integrated captive spray emitter. Peptides were separated using a 50 min gradient of 2%–30% buffer B (acetonitrile in 0.1% formic acid) with a flow rate of 250 nL/min and column temperature maintained at 50 °C.

DDA was performed in Parallel Accumulation-Serial Fragmentation (PASEF) mode to determine effective ion mobility windows for downstream diaPASEF data collection.^80^ The ddaPASEF parameters included: 100% duty cycle using accumulation and ramp times of 50 ms each, 1 TIMS-MS scan and 10 PASEF ramps per acquisition cycle. The TIMS-MS survey scan was acquired between 100 – 1700 *m/z* and 1/k0 of 0.7 – 1.3 V.s/cm^2^. Precursors with 1 – 5 charges were selected and those that reached an intensity threshold of 20,000 arbitrary units were actively excluded for 0.4 min. The quadrupole isolation width was set to 2 *m/z* for *m/z* <700 and 3 *m/z* for *m*/*z* >800, with the *m/z* between 700–800 *m/z* being interpolated linearly. The TIMS elution voltages were calibrated linearly with three points (Agilent ESI-L Tuning Mix Ions; 622, 922, 1,222 *m/z*) to determine the reduced ion mobility coefficients (1/K_0_). To perform diaPASEF, the precursor distribution in the DDA *m*/*z*-ion mobility plane was used to design an acquisition scheme for DIA data collection which included two windows in each 50 ms diaPASEF scan. Data was acquired using sixteen of these 25 Da precursor double window scans (creating 32 windows) which covered the diagonal scan line for doubly and triply charged precursors, with singly charged precursors able to be excluded by their position in the m/z-ion mobility plane. These precursor isolation windows were defined between 400 – 1200 *m/z* and 1/k0 of 0.7 – 1.3 V.s/cm^2^.

#### Immunoprecipitation mass spectrometry

For IP-MS experiments, immunoprecipitation (IP) was performed as described above (see immunoprecipitation assay). After the washing step, samples were eluted using Glycine-HCl buffer (0.2 M, pH 2.4) and rebuffered to pH 8.0. The IP eluates were reduced with 10 mM TCEP for 30 min at room temperature, and then alkylated with 15 mM iodoacetamide for 45 min at room temperature in the dark. Alkylation was quenched by the addition of 10 mM DTT. Proteins were isolated by methanol-chloroform precipitation. The protein pellets were dried and then resuspended in 50 μL 200 mM EPPS pH 8.0. The resuspended protein samples were digested with 2 μg LysC overnight at room temperature followed by the addition of 0.5 μg Trypsin for 6 h at 37°C. Protein digests were dried, resuspended in 100 μL 1% formic acid, and desalted using 10-layer C18 stage-tips before being analyzed by LC-MS.

Data were collected using an Orbitrap Exploris 480 mass spectrometer (Thermo Fisher Scientific) and coupled with a UltiMate 3000 RSLCnano System. Peptides were separated on an Aurora 25 cm × 75 μm inner diameter microcapillary column (IonOpticks), and using a 60 min linear gradient of 5 – 25% acetonitrile in 1.0% formic acid with a flow rate of 250 nL/min.

Each analysis used a TopN data-dependent method. The data were acquired using a mass range of m/z 350 – 1200, resolution 60,000, AGC target 3 × 10^6^, auto maximum injection time, dynamic exclusion of 15 sec, and charge states of 2–6. TopN 20 data-dependent MS2 spectra were acquired with a scan range starting at m/z 110, resolution 15,000, isolation window of 1.4 m/z, normalized collision energy (NCE) set at 30%, AGC target 1 × 10^5^ and the automatic maximum injection time.

### QUANTIFICATION AND STATISTICAL ANALYSIS

#### Flow-cytometry-based assays

All flow cytometry-based assays (degradation and viability assays) were run in triplicate (n=3) unless otherwise specified where n represents a distinct flow cytometric specimen (a well or tube containing ~100,000 cells). P-values were calculated using GraphPad Prism two-sided t-test and are centered around the mean and error bars represent the SEM unless otherwise specified.

#### ChIP-seq alignment and sample QC

All ChIP-seq samples were aligned to the UCSC release of the hg38.p13^81,82^ reference genome using the STAR aligner^83^ (v2.7.5b) in paired end mode with explicitly specified parameters: *winAnchorMultimapNmax=100*, *twopassMode=Basic*, *outReadsUnmapped=None*, *outSAMstrandField=intronMotif*, *outSAMunmapped=None*, *outMultimapperOrder=Random*, *outSAMmultNmax=1*, *alignIntronMax=1*, *alignEndsType=EndToEnd*, and *alignMatesGapMax=1000*. Aligned .bam files were filtered using SAMtools^84,85^ (v1.10) to initially remove mapped fragments fully contained within the ENCODE exclusion list for GRCh38^86^ (dataset: ENCFF356LFX) followed by further filtering to remove duplicate alignments and mapped fragments over 1,000 BP.

#### Normalization

In order to effectively account for/remove excess variation due to differences in sequencing depth in non-peak regions in the presence of high signal strength, particularly among GR pulldown samples, we adopted an approach integrating techniques from the TMM^87^ and Median-Ratio^88^ normalization methods. Specifically, we tabulated aligned fragments falling within bins of size 75 BP. Neighboring bins with similar length-normalized counts (typically less than 33% change) were merged to increase per-bin information; the percent-change limit implicitly differentiates peak and background bins. Following merging, sample-specific normalization scale factors were estimated from the remaining bins using the method of Median-Ratio. In brief, pseudo-counts were estimated for each remaining bin as the geometric mean across samples. Sample-specific scale factors were then calculated as the median of ratios between sample-bin-counts and bin-pseudo-counts.

#### Peak calling

To improve power to detect true peaks in the presence of replicate samples for the RARA/UBR5 experiment, replicate mapped fragments were merged together across replicates, first down-sampling the higher sequencing depth sample to the depth of the smaller to mitigate depth-related biases. Across all samples, then, condition-specific peaks were statistically assessed using MACS2^89,90^ (v2.2.7.1) using a significance cut-off of q-value <= 0.01. As simple depth-normalization does not properly remove excess variation due to sequencing depth for our data (see above), we were unable to run the standard MACS2 pipeline which uses this normalization. Instead, we implement a custom MACS2 pipeline using the package sub-commands.

In brief, we convert the ChIP fragments to a pileup in BedGraph format using *MACS2 pileup* in paired-end mode, after which the pileup is normalized by scaling with the sample-specific scale-factor (see above). The normalized ChIP track will be compared to a multiscale estimate of the background noise derived from the Input fragments from the matched experimental condition. At the fragment length level, input reads were converted to a pileup in single-end mode by extending read starts to a fragment length (median length across paired-end fragments) centered on the read start and scaled by one half to account for the double counting of the paired-end data. At the small local scale, an analogous pileup was constructed with extension length of 500BP, with a correction factor of (fragment length) / (2 * 500BP). At the large local scale, a further analogous pileup was constructed with extension length of 5,000BP, with a correction factor of (fragment length) / (2 * 5,000BP). These noise estimates were merged into a single estimate of Input noise by taking at each point the maximum of the fragment length, small local, and large local pileups, with a non-zero lower bound of the global background noise, calculated as the number of input reads multiplied by the fragment length over the effective genome size. This merged noise track was then normalized using the above-described factor.

To further reduce false positives and calls with low biological significance, the post-normalization minimum of the noise pileup was increased, if necessary, to be at least 2, implying that discovered peaks would have normalized pileup signal of significantly greater than 2. To discover such peaks, the constructed ChIP and noise pileups were compared using *MACS2 bdgcmp* with the q-value as the significance metric. Finally, peaks were called at the q-value <= 0.01 threshold using *MACS2 bdgpeakcall*.

Condition-specific peaks were merged across experimental conditions into global peak lists within either the RARA-UBR5 or the GR-UBR5 experiments respectively. In a manner similar to that used by the R package *DiffBind*^91^ peak ranges are pooled across conditions with overlapping peaks merged into single, typically longer, peaks.

#### ChIP-seq peak statistical analysis

Merged peaks were annotated against genomic region and associated gene using the *Chipseeker*^92^ (v1.32.0) R package referencing the R database *TxDb.Hsapiens.UCSC.hg38.knownGenexDb* (v3.15.0). Following annotation, fragment counts within peaks were tested for differential binding (DB) between experimental conditions using the R package *DESeq2*^88,93^ (v2.1.36.0) with custom normalization factors as described above. Test significance was corrected for false sign rate (analogous to but broadly stricter than false discovery rate) using the R package *ashr*^94^ (v2.5.54).

Merged peak ranges were then converted into .fasta formatted sequences using the R package *Biostrings* (v2.64.0) referencing the *BSgenome.Hsapiens.UCSC.hg38* (v1.4.4) reference R database. Sequences were analyzed for motifs using the online portal to the MEME Suite^95^ (v5.4.1) of tools, referencing *STREME*^96^ for *de novo* motif discovery and *SEA*^97^ for motif enrichment referencing the JASPAR CORE 2022, non-redundant, vertebrate database.^98^

#### RNA sequencing analysis

Paired-end reads were aligned to the hg38.p13^81,82^ reference genome with the STAR^83^ (v5.2.7b) aligner as part of the RSEM^99^ command line tool. Gene transcript abundance was further quantified from aligned fragments using RSEM to generate a gene-by-sample matrix of expected counts. Differential expression was tested, as with the ChIP-seq data, using the *DESeq*^88,93^*2* and ashr^94^ pipeline, this time calculating sample-specific scale factor offset using the method of median-ratio as implemented in the R package *EBSeq*^100^ (v1.36.0). For plotting in heatmaps, genes were selected based on ChIP-seq peak annotation and subset.

#### LC-MS data analysis (IP-MS)

Proteome Discoverer 2.4 (Thermo Fisher Scientific) was used for .RAW file processing and controlling peptide and protein level false discovery rates, assembling proteins from peptides, and protein quantification from peptides. MS/MS spectra were searched against a Uniprot human database (January 2021) with both the forward and reverse sequences as well as known contaminants such as human keratins. Database search criteria were as follows: tryptic with two missed cleavages, a precursor mass tolerance of 10 ppm, fragment ion mass tolerance of 0.6 Da, static alkylation of cysteine (57.02146 Da) and variable oxidation of methionine (15.99491 Da). Peptides were quantified using the MS1 Intensity, and peptide abundance values were summed to yield the protein abundance values.

Resulting data was filtered to only include proteins that had a minimum of 2 abundance counts in at least two runs. Abundances were normalized and scaled using in-house scripts in the R framework. Missing values in the dataset were imputed by random selection from a gaussian distribution centered around the mean of the existing data and with the mean relative standard deviation of the dataset. Significant changes comparing the relative protein abundance between samples were assessed by moderated t test as implemented in the limma package within the R framework.

#### LC-MS data analysis (whole proteomics)

The diaPASEF raw file processing and controlling peptide and protein level false discovery rates, assembling proteins from peptides, and protein quantification from peptides was performed using library free analysis in DIA-NN 1.8.^101^ Library free mode performs an in silico digestion of a given protein sequence database alongside deep learning-based predictions to extract the DIA precursor data into a collection of MS2 spectra. The search results are then used to generate a spectral library which is then employed for the targeted analysis of the DIA data searched against a Swissprot human database (January 2021). Database search criteria largely followed the default settings for directDIA including: tryptic with two missed cleavages, carbomidomethylation of cysteine, and oxidation of methionine and precursor Q-value (FDR) cut-off of 0.01. Precursor quantification strategy was set to Robust LC (high accuracy) with RT-dependent cross run normalization. Proteins with missing values in any of the treatments and with poor quality data were excluded from further analysis (summed abundance across channels of <100 and mean number of precursors used for quantification <2). Protein abundances were scaled using in-house scripts in the R framework^102^ and statistical analysis was carried out using the limma package within the R framework.^103^

All other statistical details of experiments can be found in the results section, figure legends, and corresponding methods.

## Supplementary Material

Supplementary Material

## Figures and Tables

**KEY RESOURCES TABLE:** 

REAGENT or RESOURCE	SOURCE	IDENTIFIER

Antibodies		

Goat anti-mouse alexa fluor 790	Thermo Fisher	Cat# A11357; RRID:AB_2534140
Mouse anti-strep	IBA lifesciences	Cat# 2-1507-001; RRID:AB_513133
Mouse anti-his	Sigma	Cat# SAB1305538; RRID:AB_2687993
Rabbit anti-UBR5	CST	Clone: D608Z; RRID:AB_2799679
Rabbit anti-RARA	CST	Clone: E6Z6K; RRID:AB_2799625
Rabbit anti-NR3C1	CST	Clone: D6H2L; RRID:AB_2631286
Rabbit anti-SRC1	CST	Clone: 128E7; RRID:AB_2196189
Rabbit anti-SRC2	CST	Clone: D2X4M; RRID:AB_2800266
Mouse anti-B-Actin	CST	Clone: 3700; RRID:AB_2242334
Mouse anti-Histone H3	CST	Clone: 9715; RRID:AB_331563
Goat anti-Mouse 800CW	LI-COR Biosciences	CAT#: 926-32211; RRID:AB_621842
Goat anti-Rabbit 680LT	LI-COR Biosciences	CAT#: 925-68021; RRID:AB_2713919
Anti-RARA	Diagenode	CAT#: C15310155
Anti-GR	Diagenode	CAT#: C15200010; RRID:AB_2801409
Anti-GR	Santa Cruz	Clone: G-5; RRID:AB_2687823

Bacterial and virus strains		

E. coli BL21-CodonPlus(DE3)-RIL	Agilent	Cat# 230245
NEB Stable Competent *E. coli*	NEB	Cat# C3040H
NEB 5-alpha Competent *E. coli*	NEB	Cat# C2987H

Chemicals, peptides, and recombinant proteins		

Protease inhibitor cocktail	Sigma	Cat# S8830
9-cis-retinoic acid	abcr	Cat# AB348741
all-trans-retinoic acid	Sigma	Cat# R2625
BMS-614	Tocris	Cat # 3660
dexamethasone	Sigma	Cat# D1756
beta-Estradiol	Sigma	Cat# E8875
fulvestrant	Sigma	Cat# I4409
4-hydroxytamoxifen	Sigma	Cat# H7904
GDC-0927	MedChemExpress	Cat#: HY-111484
Elacestrant	MedChemExpress	Cat#: HY-19822
Giredestrant	MedChemExpress	Cat#: HY-109176
Camizestrant	MedChemExpress	Cat#: HY-136255
Amcenestrant	MedChemExpress	Cat#: HY-133017
AZD-9496	MedChemExpress	Cat#: HY-12870
Brilanestrant	MedChemExpress	Cat#: HY-12864
LSZ-102	MedChemExpress	Cat#: HY-111486
ARV-471	MedChemExpress	Cat#: HY-138642
ERD-308	MedChemExpress	Cat#: HY-128600
Calcitriol	MedChemExpress	Cat# HY-10002
Recombinant E2, UbcH7	R&D systems	Cat# E2-640-100
Recombinant E1, UBE1	R&D systems	Cat# E-305
Recombinant Ubiquitin-IRdye680maleimide	This study	N/A
Recombinant Ubiquitin	R&D systems	Cat# U-100H
SF4 Baculo-Express Media	BioConcept	Cat# 900F38
3xFLAG peptide	Sigma	Cat# F4799
Fluorescein NCOA1 peptide aa687-698 FAM-ARHKILHRLLQEGS	Peptides & Elephants	N/A
UBR5 aa244-256 PGEDLMSLLDADI	Peptides & Elephants	N/A
UBR5 aa1098-1110 LQPYLRELLSAKD	Peptides & Elephants	N/A
UBR5 aa1251-1263 RLDLLYRLLTATN	Peptides & Elephants	N/A
UBR5 aa1366-1378 ASSRIGHLLPEEQ	Peptides & Elephants	N/A
UBR5 aa1325-1337 AQLALERVLQDWN	Peptides & Elephants	N/A
UBR5 aa1394-1406 DILLLDTLLGTLV	Peptides & Elephants	N/A
UBR5 aa1794-1806 QISDLMGLIPKYN	Peptides & Elephants	N/A
UBR5 aa2575-2587 MYESLRQLILASQ	Peptides & Elephants	N/A

Deposited data		

UBR5 structure model (tetramer, WT)	This study	PDB: 8P83
UBR5 structure model (dimer, Δaa522-720)	This study	PDB: 8P82
UBR5 structure map (tetramer, WT)	This study	EMDB: 17540
UBR5 structure map (dimer, Δaa522-720)	This study	EMDB: 17539
UBR5~RARA/RXRA negative stain density map	This study	EMDB: 17542
Whole proteomics (ATRA treatment)	This study	PXD040953
IPMS (RARA Pulldown)	This study	PXD041749
Superseries (RARA/UBR5)	This study	GSE213795
ChIPseq (GR/UBR5)	This study	GSE213742
RNAseq (ATRA)	This study	GSE213793
RNAseq (Dexamethasone)	This study	GSE213793

Experimental models: Cell lines		

U937-Cas9	Broad GPP	N/A
NB4-Cas9	Broad GPP	N/A
A549	ATCC	CRM-CCL-185
HEK293T	Broad GPP	N/A

Experimental models: Organisms/strains		

E. coli BL21-CodonPlus(DE3)-RIL	Agilent	Cat# 230245
Sf9 Insect cells	Thermo Fisher	Cat# 11496015
High-Five Insect cells	Thermo Fisher	Cat# B85502
Expi293F cells	Thermo Fisher	Cat #A14527

Oligonucleotides		

Fluorescein-RARE forward/56-FAM/CTCCGGTTCACCGAAAGTTCATAG	IDT	N/A
RARE complement CTATGAACTTTCGGTGAACCGGAG	IDT	N/A

Recombinant DNA		

pDEST_FLAG-UBR5	This study	N/A
pDEST_FLAG-UBR5Δ83-347 (ΔUBA)	This study	N/A
pDEST_FLAG-UBR5 (V196K)	This study	N/A
pDEST_FLAG-UBR5Δ522-720 (Δtandem SBB)	This study	N/A
pDEST_FLAG-UBR5Δ1181-1243 (ΔUBR)	This study	N/A
pDEST_FLAG-UBR5Δ2218-2798 (ΔHECT)	This study	N/A
pDEST_FLAG-UBR5Δ522-720,Δ2218-2798 (Δtandem SBB, ΔHECT)	This study	N/A
pAC8_Strep-Smt3-TEV-NCOA1	This study	N/A
pET28a_His-Smt3-TEV-RARA aa175-421 (LBD)	This study	N/A
pET28a_His-Smt3-TEV-RARA aa175-421,V240E (LBD)	This study	N/A
pET28a_His-Smt3-TEV-RARA aa83-421 (DBD-LBD)	This study	N/A
pET28a_His-Smt3-TEV-RXRA aa224-462 (LBD)	This study	N/A
pET28a_His-Smt3-TEV-RXRA aa224-462, V280E (LBD)	This study	N/A
pET28a_His-Smt3-TEV-RXRA aa128-462 (DBD-LBD)	This study	N/A
pET28a_His-Smt3-TEV-ER aa301-552 (LBD)	This study	N/A
pET28a_His-Smt3-TEV-VDR aa118-427 (DBD-LBD)	This study	N/A
pZucchini2_SFFV-RARA-eGFP-IRES-mCherry	This study	N/A
pSquash2_SFFV-eGFP- NR3C1-IRES-mCherry	This study	N/A
pZucchini2_SFFV-RXRA-eGFP-IRES-mCherry	This study	N/A
pZucchini2_SFFV-ESR1-eGFP-IRES-mCherry	This study	N/A
pZucchini2_SFFV-NR1l1-eGFP-IRES-mCherry	This study	N/A
pZucchini2_SFFV-NR3C3-eGFP-IRES-mCherry	This study	N/A
pZucchini2_SFFV-NR3C4-eGFP-IRES-mCherry	This study	N/A
pZucchini2_SFFV-NR1H3-eGFP-IRES-mCherry	This study	N/A
pZucchini2_SFFV-NR1A2-eGFP-IRES-mCherry	This study	N/A

Software and algorithms		

FEI EPU v2.7.0	Thermo Scientific	https://www.thermofisher.com/
cryoFLARE	Schenk et al.^62^	https://www.cryoflare.org/
cryoSPARC v3-v4	Punjani et al.^63^	https://cryosparc.com/
LocScale	Jakobi et al.^64^	https://git.embl.de/jakobi/LocScale
AlphaFold v2	Jumper et al.^65^	https://github.com/deepmind/alphafold
Isolde v1.3	Croll^66^	https://isolde.cimr.cam.ac.uk/
Phenix v1.20	Afonine et al.^67^	https://phenix-online.org/
ChimeraX v1.3	Pettersen et al.^68^	https://www.rbvi.ucsf.edu/chimerax/
PyMol v2.3.3	Schrodinger, LLC	https://pymol.org/2/
Rosetta	Wang et al.^69^	https://www.rosettacommons.org/
coot v0.9.6	Emsley et al.^70^	https://www2.mrc-lmb.cam.ac.uk/personal/pemsley/coot/

Other		

HisTrap FF	Cytiva	Cat# 17525501
Mono Q 5/50 GL	Cytiva	Cat# 17516601
Superose 6 10/300 Increase	Cytiva	Cat# 29091596
Expi293 Expression System Kit	Thermo Fisher	Cat# A14635
FLAG M2 gel	Sigma	Cat# A2220
Formvar/Carbon 300 mesh copper grids	Ted Pella	Cat# 01753-F
GFP-Trap Magnetic Agarose beads	ChromoTek	Cat#: GTMA-20

## Highlights

- Genetic screens identify UBR5 as a pan-regulator of NR stability
- Agonist-bound NRs promote competition between NCOA and UBR5 complexes
- Cryo-EM structure of full-length UBR5 reveals a homotetrameric ring architecture
- UBR5 targets NRs directly on chromatin and effects transcriptional output

## References

[1] Ismail A, and Nawaz Z (2005). Nuclear hormone receptor degradation and gene transcription: an update. IUBMB Life 57, 483–490. 10.1080/15216540500147163. 16081369

[2] Wang L, Oh TG, Magida J, Estepa G, Obayomi SMB, Chong LW, Gatchalian J, Yu RT, Atkins AR, Hargreaves D, (2021). Bromodomain containing 9 (BRD9) regulates macrophage inflammatory responses by potentiating glucocorticoid receptor activity. Proc. Natl. Acad. Sci. USA 118, e2109517118. 10.1073/pnas.2109517118. 34446564 PMC8536317

[3] Fu T, Coulter S, Yoshihara E, Oh TG, Fang S, Cayabyab F, Zhu Q, Zhang T, Leblanc M, Liu S, (2019). FXR regulates intestinal cancer stem cell proliferation. Cell 176, 1098–1112.e18. 10.1016/j.cell.2019.01.036. 30794774 PMC6701863

[4] Ding N, Yu RT, Subramaniam N, Sherman MH, Wilson C, Rao R, Leblanc M, Coulter S, He M, Scott C, (2013). A vitamin D receptor/SMAD genomic circuit gates hepatic fibrotic response. Cell 153, 601–613. 10.1016/j.cell.2013.03.028. 23622244 PMC3673534

[5] Huang P, Chandra V, and Rastinejad F (2010). Structural overview of the nuclear receptor superfamily: insights into physiology and therapeutics. Annu. Rev. Physiol. 72, 247–272. 10.1146/annurev-physiol-021909-135917. 20148675 PMC3677810

[6] Zhu J, Gianni M, Kopf E, Honoré N, Chelbi-Alix M, Koken M, Quignon F, Rochette-Egly C, and de Thé H (1999). Retinoic acid induces proteasome-dependent degradation of retinoic acid receptor a (RARa) and oncogenic RARa fusion proteins. Proc. Natl. Acad. Sci. USA 96, 14807–14812. 10611294 10.1073/pnas.96.26.14807 PMC24729

[7] Dace A, Zhao L, Park KS, Furuno T, Takamura N, Nakanishi M, West BL, Hanover JA, and Cheng S (2000). Hormone binding induces rapid proteasome-mediated degradation of thyroid hormone receptors. Proc. Natl. Acad. Sci. USA 97, 8985–8990. 10.1073/pnas.160257997. 10908671 PMC16808

[8] Wallace AD, and Cidlowski JA (2001). Proteasome-mediated glucocorticoid receptor degradation restricts transcriptional signaling by glucocorticoids. J. Biol. Chem. 276, 42714–42721. 10.1074/jbc.M106033200. 11555652

[9] Nawaz Z, Lonard DM, Dennis AP, Smith CL, and O’Malley BW (1999). Proteasome-dependent degradation of the human estrogen receptor. Proc. Natl. Acad. Sci. USA 96, 1858–1862. 10051559 10.1073/pnas.96.5.1858 PMC26701

[10] Giguère V, and Evans RM (2022). Chronicle of a discovery: the retinoic acid receptor. J. Mol. Endocrinol. 69, T1–T11. 10.1530/JME-22-0117. 35900848

[11] Zhang XW, Yan XJ, Zhou ZR, Yang FF, Wu ZY, Sun HB, Liang WX, Song AX, Lallemand-Breitenbach V, Jeanne M, (2010). Arsenic trioxide controls the fate of the PML-RARalpha oncoprotein by directly binding PML. Science 328, 240–243. 10.1126/science.1183424. 20378816

[12] Jimenez JJ, Chale RS, Abad AC, and Schally AV (2020). Acute promyelocytic leukemia (APL): a review of the literature. Oncotarget 11, 992–1003. 10.18632/oncotarget.27513. 32215187 PMC7082115

[13] Martens JH, Brinkman AB, Simmer F, Francoijs KJ, Nebbioso A, Ferrara F, Altucci L, and Stunnenberg HG (2010). PML-RARalpha/RXR alters the epigenetic landscape in acute promyelocytic leukemia. Cancer Cell 17, 173–185. 10.1016/j.ccr.2009.12.042. 20159609

[14] Nasr R, Guillemin MC, Ferhi O, Soilihi H, Peres L, Berthier C, Rousselot P, Robledo-Sarmiento M, Lallemand-Breitenbach V, Gourmel B, (2008). Eradication of acute promyelocytic leukemia-initiating cells through PML-RARA degradation. Nat. Med. 14, 1333–1342. 10.1038/nm.1891. 19029980

[15] Ablain J, Leiva M, Peres L, Fonsart J, Anthony E, and de Thé H (2013). Uncoupling RARA transcriptional activation and degradation clarifies the bases for APL response to therapies. J. Exp. Med. 210, 647–653. 10.1084/jem.20122337. 23509325 PMC3620357

[16] Long X, and Nephew KP (2006). Fulvestrant (ICI 182,780)-dependent interacting proteins mediate immobilization and degradation of estrogen receptor-alpha. J. Biol. Chem. 281, 9607–9615. 10.1074/jbc.M510809200. 16459337

[17] Guan J, Zhou W, Hafner M, Blake RA, Chalouni C, Chen IP, De Bruyn T, Giltnane JM, Hartman SJ, Heidersbach A, (2019). Therapeutic ligands antagonize estrogen receptor function by impairing its mobility. Cell 178, 949–963.e18. 10.1016/j.cell.2019.06.026. 31353221

[18] Kargbo RB (2021). Androgen receptor protein degradation in the treatment of castration-resistant prostate cancer. ACS Med. Chem. Lett. 12, 318–319. 10.1021/acsmedchemlett.1c00059. PMC7957932 33738051

[19] Ponnusamy S, Coss CC, Thiyagarajan T, Watts K, Hwang DJ, He Y, Selth LA, McEwan IJ, Duke CB, Pagadala J, (2017). Novel selective agents for the degradation of androgen receptor variants to treat castration-resistant prostate cancer. Cancer Res. 77, 6282–6298. 10.1158/0008-5472.CAN-17-0976. 28978635 PMC5890913

[20] Salami J, Alabi S, Willard RR, Vitale NJ, Wang J, Dong H, Jin M, McDonnell DP, Crew AP, Neklesa TK, and Crews CM (2018). Androgen receptor degradation by the proteolysis-targeting chimera ARCC-4 outperforms enzalutamide in cellular models of prostate cancer drug resistance. Commun. Biol. 1, 100. 10.1038/s42003-018-0105-8. 30271980 PMC6123676

[21] Jin L, and Li Y (2010). Structural and functional insights into nuclear receptor signaling. Adv. Drug Deliv. Rev. 62, 1218–1226. 10.1016/j.addr.2010.08.007. 20723571 PMC2991384

[22] S1abicki M, Kozicka Z, Petzold G, Li YD, Manojkumar M, Bunker RD, Donovan KA, Sievers QL, Koeppel J, Suchyta D, (2020). The CDK inhibitor CR8 acts as a molecular glue degrader that depletes cyclin K. Nature 585, 293–297. 10.1038/s41586-020-2374-x. 32494016 PMC7486275

[23] S1abicki M, Yoon H, Koeppel J, Nitsch L, Roy Burman SS, Di Genua C, Donovan KA, Sperling AS, Hunkeler M, Tsai JM, (2020). Small-molecule-induced polymerization triggers degradation of BCL6. Nature 588, 164–168. 10.1038/s41586-020-2925-1. 33208943 PMC7816212

[24] Wang T, Birsoy K, Hughes NW, Krupczak KM, Post Y, Wei JJ, Lander ES, and Sabatini DM (2015). Identification and characterization of essential genes in the human genome. Science 350, 1096–1101. 10.1126/science.aac7041. 26472758 PMC4662922

[25] Yu VC, Delsert C, Andersen B, Holloway JM, Devary OV, Näär AM, Kim SY, Boutin JM, Glass CK, and Rosenfeld MG (1991). RXR beta: a coregulator that enhances binding of retinoic acid, thyroid hormone, and vitamin D receptors to their cognate response elements. Cell 67, 1251–1266. 1662118 10.1016/0092-8674(91)90301-e

[26] Weikum ER, Liu X, and Ortlund EA (2018). The nuclear receptor superfamily: A structural perspective. Protein Sci. 27, 1876–1892. 10.1002/pro.3496. 30109749 PMC6201731

[27] Hurt DE, Suzuki S, Mayama T, Charmandari E, and Kino T (2016). Structural analysis on the pathologic mutant glucocorticoid receptor ligand-binding domains. Mol. Endocrinol. 30, 173–188. 10.1210/me.2015-1177. 26745667 PMC4792232

[28] Leo C, Yang X, Liu J, Li H, and Chen JD (2001). Role of retinoid receptor coactivator pockets in cofactor recruitment and transcriptional regulation. J. Biol. Chem. 276, 23127–23134. 10.1074/jbc.M100462200. 11274211

[29] Hsu JY, Fulco CP, Cole MA, Canver MC, Pellin D, Sher F, Farouni R, Clement K, Guo JA, Biasco L, (2018). CRISPR-SURF: discovering regulatory elements by deconvolution of CRISPR tiling screen data. Nat. Methods 15, 992–993. 10.1038/s41592-018-0225-6. 30504875 PMC6620603

[30] Matta-Camacho E, Kozlov G, Menade M, and Gehring K (2012). Structure of the HECT C-lobe of the UBR5 E3 ubiquitin ligase. Acta Crystallogr. Sect. F Struct. Biol. Cryst. Commun. 68, 1158–1163. 10.1107/S1744309112036937. PMC3497971 23027739

[31] Kozlov G, Nguyen L, Lin T, De Crescenzo G, Park M, and Gehring K (2007). Structural basis of ubiquitin recognition by the ubiquitin-associated (UBA) domain of the ubiquitin ligase EDD. J. Biol. Chem. 282, 35787–35795. 10.1074/jbc.M705655200. 17897937

[32] Muñoz-Escobar J, Matta-Camacho E, Kozlov G, and Gehring K (2015). The MLLE domain of the ubiquitin ligase UBR5 binds to its catalytic domain to regulate substrate binding. J. Biol. Chem. 290, 22841–22850. 10.1074/jbc.M115.672246. 26224628 PMC4566254

[33] Swenson SA, Gilbreath TJ, Vahle H, Hynes-Smith RW, Graham JH, Law HC, Amador C, Woods NT, Green MR, and Buckley SM (2020). UBR5 HECT domain mutations identified in mantle cell lymphoma control maturation of B cells. Blood 136, 299–312. 10.1182/blood.2019002102. 32325489 PMC7365918

[34] Kamadurai HB, Souphron J, Scott DC, Duda DM, Miller DJ, Stringer D, Piper RC, and Schulman BA (2009). Insights into ubiquitin transfer cascades from a structure of a UbcH5B approximately ubiquitin-HECT(NEDD4L) complex. Mol. Cell 36, 1095–1102. 10.1016/j.molcel.2009.11.010. 20064473 PMC2859195

[35] Hunkeler M, Jin CY, Ma MW, Monda JK, Overwijn D, Bennett EJ, and Fischer ES (2021). Solenoid architecture of HUWE1 contributes to ligase activity and substrate recognition. Mol. Cell 81, 3468–3480.e7. 10.1016/j.molcel.2021.06.032. 34314700 PMC8476073

[36] Nair RM, Seenivasan A, Liu B, Chen D, Lowe ED, and Lorenz S (2021). Reconstitution and structural analysis of a HECT ligase-ubiquitin complex via an activity-based probe. ACS Chem. Biol. 16, 1615–1621. 10.1021/acschembio.1c00433. PMC8453484 34403242

[37] Maspero E, Mari S, Valentini E, Musacchio A, Fish A, Pasqualato S, and Polo S (2011). Structure of the HECT:ubiquitin complex and its role in ubiquitin chain elongation. EMBO Rep. 12, 342–349. 10.1038/embor.2011.21. 21399620 PMC3077247

[38] Kamadurai HB, Qiu Y, Deng A, Harrison JS, Macdonald C, Actis M, Rodrigues P, Miller DJ, Souphron J, Lewis SM, (2013). Mechanism of ubiquitin ligation and lysine prioritization by a HECT E3. eLife 2, e00828. 10.7554/eLife.00828. 23936628 PMC3738095

[39] Ohtake F, Tsuchiya H, Saeki Y, and Tanaka K (2018). K63 ubiquitylation triggers proteasomal degradation by seeding branched ubiquitin chains. Proc. Natl. Acad. Sci. USA 115, E1401–E1408. 10.1073/pnas.1716673115. 29378950 PMC5816176

[40] Yau RG, Doerner K, Castellanos ER, Haakonsen DL, Werner A, Wang N, Yang XW, Martinez-Martin N, Matsumoto ML, Dixit VM, and Rape M (2017). Assembly and function of heterotypic ubiquitin chains in cell-cycle and protein quality control. Cell 171, 918–933.e20. 10.1016/j.cell.2017.09.040. 29033132 PMC5669814

[41] Balmer JE, and Blomhoff R (2002). Gene expression regulation by retinoic acid. J. Lipid Res. 43, 1773–1808. 10.1194/jlr.r100015-jlr200. 12401878

[42] Yang L, Zhao H, Li SW, Ahrens K, Collins C, Eckenrode S, Ruan QG, McIndoe RA, and She JX (2003). Gene expression profiling during all-trans retinoic acid-induced cell differentiation of acute promyelocytic leukemia cells. J. Mol. Diagn. 5, 212–221. 10.1016/S1525-1578(10)60476-X. 14573779 PMC1907337

[43] Lonard DM, Nawaz Z, Smith CL, and O’Malley BW (2000). The 26S proteasome is required for estrogen receptor-alpha and coactivator turnover and for efficient estrogen receptor-alpha transactivation. Mol. Cell 5, 939–948. 10.1016/s1097-2765(00)80259-2. 10911988

[44] Savory JG, Hsu B, Laquian IR, Giffin W, Reich T, Haché RJ, and Lefebvre YA (1999). Discrimination between NL1- and NL2-mediated nuclear localization of the glucocorticoid receptor. Mol. Cell. Biol. 19, 1025–1037. 10.1128/MCB.19.2.1025. 9891038 PMC116033

[45] Stahn C, Löwenberg M, Hommes DW, and Buttgereit F (2007). Molecular mechanisms of glucocorticoid action and selective glucocorticoid receptor agonists. Mol. Cell. Endocrinol. 275, 71–78. 10.1016/j.mce.2007.05.019. 17630118

[46] Masuyama H, and MacDonald PN (1998). Proteasome-mediated degradation of the vitamin D receptor (VDR) and a putative role for SUG1 interaction with the AF-2 domain of VDR. J. Cell. Biochem. 71, 429–440. 9831079

[47] Lange CA, Shen T, and Horwitz KB (2000). Phosphorylation of human progesterone receptors at serine-294 by mitogen-activated protein kinase signals their degradation by the 26S proteasome. Proc. Natl. Acad. Sci. USA 97, 1032–1037. 10655479 10.1073/pnas.97.3.1032 PMC15511

[48] Tyagi RK, Lavrovsky Y, Ahn SC, Song CS, Chatterjee B, and Roy AK (2000). Dynamics of intracellular movement and nucleocytoplasmic recycling of the ligand-activated androgen receptor in living cells. Mol. Endocrinol. 14, 1162–1174. 10.1210/mend.14.8.0497. 10935541

[49] Yeap BB, Krueger RG, and Leedman PJ (1999). Differential post-transcriptional regulation of androgen receptor gene expression by androgen in prostate and breast cancer cells. Endocrinology 140, 3282–3291. 10.1210/endo.140.7.6769. 10385425

[50] Lee DK, and Chang C (2003). Endocrine mechanisms of disease: expression and degradation of androgen receptor: mechanism and clinical implication. J. Clin. Endocrinol. Metab. 88, 4043–4054. 10.1210/jc.2003-030261. 12970260

[51] Hur E, Pfaff SJ, Payne ES, Grøn H, Buehrer BM, and Fletterick RJ (2004). Recognition and accommodation at the androgen receptor coactivator binding interface. PLoS Biol. 2, E274. 10.1371/journal.pbio.0020274. 15328534 PMC509409

[52] Yu X, Yi P, Hamilton RA, Shen H, Chen M, Foulds CE, Mancini MA, Ludtke SJ, Wang Z, and O’Malley BW (2020). Structural in-sights of transcriptionally active, full-length androgen receptor coactivator complexes. Mol. Cell 79, 812–823.e4. 10.1016/j.molcel.2020.06.031. 32668201 PMC7483370

[53] Bidard FC, Kaklamani VG, Neven P, Streich G, Montero AJ, Forget F, Mouret-Reynier MA, Sohn JH, Taylor D, Harnden KK, (2022). Elacestrant (oral selective estrogen receptor degrader) versus Standard Endocrine Therapy for estrogen receptor-Positive, Human epidermal growth factor Receptor 2-Negative Advanced Breast Cancer: results from the Randomized Phase III EMERALD Trial. J. Clin. Oncol. 40, 3246–3256. 10.1200/JCO.22.00338. 35584336 PMC9553388

[54] Pike AC, Brzozowski AM, Walton J, Hubbard RE, Thorsell AG, Li YL, Gustafsson JA, and Carlquist M (2001). Structural insights into the mode of action of a pure antiestrogen. Structure 9, 145–153. 10.1016/s0969-2126(01)00568-8. 11250199

[55] Wardell SE, Marks JR, and McDonnell DP (2011). The turnover of estrogen receptor alpha by the selective estrogen receptor degrader (SERD) fulvestrant is a saturable process that is not required for antagonist efficacy. Biochem. Pharmacol. 82, 122–130. 10.1016/j.bcp.2011.03.031. 21501600 PMC3109090

[56] Bolt MJ, Stossi F, Callison AM, Mancini MG, Dandekar R, and Mancini MA (2015). Systems level-based RNAi screening by high content analysis identifies UBR5 as a regulator of estrogen receptor-alpha protein levels and activity. Oncogene 34, 154–164. 10.1038/onc.2013.550. 24441042 PMC4871123

[57] Ong SS, Goktug AN, Elias A, Wu J, Saunders D, and Chen T (2014). Stability of the human pregnane X receptor is regulated by E3 ligase UBR5 and serine/threonine kinase DYRK2. Biochem. J. 459, 193–203. 10.1042/BJ20130558. 24438055 PMC3959618

[58] Schukur L, Zimmermann T, Niewoehner O, Kerr G, Gleim S, Bauer-Probst B, Knapp B, Galli GG, Liang X, Mendiola A, (2020). Identification of the HECT E3 ligase UBR5 as a regulator of MYC degradation using a CRISPR/Cas9 screen. Sci. Rep. 10, 20044. 10.1038/s41598-020-76960-z. 33208877 PMC7676242

[59] Qiao X, Liu Y, Prada ML, Mohan AK, Gupta A, Jaiswal A, Sharma M, Merisaari J, Haikala HM, Talvinen K, (2020). UBR5 is coamplified with MYC in breast tumors and encodes an ubiquitin ligase that limits MYC-dependent apoptosis. Cancer Res. 80, 1414–1427. 10.1158/0008-5472.CAN-19-1647. 32029551

[60] Kaisari S, Miniowitz-Shemtov S, Sitry-Shevah D, Shomer P, Kozlov G, Gehring K, and Hershko A (2022). Role of ubiquitin-protein ligase UBR5 in the disassembly of mitotic checkpoint complexes. Proc. Natl. Acad. Sci. USA 119, e2121478119. 10.1073/pnas.2121478119. 35217622 PMC8892521

[61] Mark KG, Kolla S, Aguirre AG, Garshott DM, Schmitt S, Haakonsen DL, Xu C, Kater L, Kempf G, Martinez-Gonzalez B, (2023). Orphan quality control shapes network dynamics and gene expression. Cell 186. 10.1016/j.cell.2023.06.015. 37478862

[62] Schenk AD, Cavadini S, Thomä NH, and Genoud C (2020). Live analysis and reconstruction of single-particle cryo-electron microscopy data with CryoFLARE. J. Chem. Inf. Model. 60, 2561–2569. 10.1021/acs.jcim.9b01102. 32233514

[63] Punjani A, Rubinstein JL, Fleet DJ, and Brubaker MA (2017). cryoSPARC: algorithms for rapid unsupervised cryo-EM structure determination. Nat. Methods 14, 290–296. 10.1038/nmeth.4169. 28165473

[64] Jakobi AJ, Wilmanns M, and Sachse C (2017). Model-based local density sharpening of cryo-EM maps. eLife 6, e27131. 10.7554/eLife.27131. 29058676 PMC5679758

[65] Jumper J, Evans R, Pritzel A, Green T, Figurnov M, Ronneberger O, Tunyasuvunakool K, Bates R, Žídek A, Potapenko A, (2021). Highly accurate protein structure prediction with AlphaFold. Nature 596, 583–589. 10.1038/s41586-021-03819-2. 34265844 PMC8371605

[66] Croll TI (2018). Isolde: a physically realistic environment for model building into low-resolution electron-density maps. Acta Crystallogr. D Struct. Biol. 74, 519–530. 10.1107/S2059798318002425. 29872003 PMC6096486

[67] Afonine PV, Poon BK, Read RJ, Sobolev OV, Terwilliger TC, Urzhumtsev A, and Adams PD (2018). Real-space refinement in PHENIX for cryo-EM and crystallography. Acta Crystallogr. D Struct. Biol. 74, 531–544. 10.1107/S2059798318006551. 29872004 PMC6096492

[68] Pettersen EF, Goddard TD, Huang CC, Meng EC, Couch GS, Croll TI, Morris JH, and Ferrin TE (2021). UCSF ChimeraX: structure visualization for researchers, educators, and developers. Protein Sci. 30, 70–82. 10.1002/pro.3943. 32881101 PMC7737788

[69] Wang RY, Song Y, Barad BA, Cheng Y, Fraser JS, and DiMaio F (2016). Automated structure refinement of macromolecular assemblies from cryo-EM maps using Rosetta. eLife 5, e17219. 10.7554/eLife.17219. 27669148 PMC5115868

[70] Emsley P, Lohkamp B, Scott WG, and Cowtan K (2010). Features and development of coot. Acta Crystallogr. D Biol. Crystallogr. 66, 486–501. 10.1107/S0907444910007493. 20383002 PMC2852313

[71] Pogenberg V, Guichou JF, Vivat-Hannah V, Kammerer S, Pérez E, Germain P, de Lera AR, Gronemeyer H, Royer CA, and Bourguet W (2005). Characterization of the interaction between retinoic acid receptor/retinoid X receptor (RAR/RXR) heterodimers and transcriptional coactivators through structural and fluorescence anisotropy studies. J. Biol. Chem. 280, 1625–1633. 10.1074/jbc.M409302200. 15528208

[72] Marks BD, Qadir N, Eliason HC, Shekhani MS, Doering K, and Vogel KW (2005). Multiparameter analysis of a screen for progesterone receptor ligands: comparing fluorescence lifetime and fluorescence polarization measurements. Assay Drug Dev. Technol. 3, 613–622. 10.1089/adt.2005.3.613. 16438657

[73] Stark H (2010). GraFix: stabilization of fragile macromolecular complexes for single particle cryo-EM. Methods Enzymol. 481, 109–126. 10.1016/S0076-6879(10)81005-5. 20887855

[74] Scheres SH (2012). RELION: implementation of a Bayesian approach to cryo-EM structure determination. J. Struct. Biol. 180, 519–530. 10.1016/j.jsb.2012.09.006. 23000701 PMC3690530

[75] Bryant P, Pozzati G, and Elofsson A (2022). Author Correction: improved prediction of protein-protein interactions using AlphaFold2. Nat. Commun. 13, 1694. 10.1038/s41467-022-29480-5. 35332153 PMC8948285

[76] Afonine PV, Klaholz BP, Moriarty NW, Poon BK, Sobolev OV, Terwilliger TC, Adams PD, and Urzhumtsev A (2018). New tools for the analysis and validation of cryo-EM maps and atomic models. Acta Crystallogr. D Struct. Biol. 74, 814–840. 10.1107/S2059798318009324. 30198894 PMC6130467

[77] Williams CJ, Headd JJ, Moriarty NW, Prisant MG, Videau LL, Deis LN, Verma V, Keedy DA, Hintze BJ, Chen VB, (2018). MolProbity: more and better reference data for improved all-atom structure validation. Protein Sci. 27, 293–315. 10.1002/pro.3330. 29067766 PMC5734394

[78] Barad BA, Echols N, Wang RY, Cheng Y, DiMaio F, Adams PD, and Fraser JS (2015). EMRinger: side chain-directed model and map validation for 3D cryo-electron microscopy. Nat. Methods 12, 943–946. 10.1038/nmeth.3541. 26280328 PMC4589481

[79] Donovan KA, An J, Nowak RP, Yuan JC, Fink EC, Berry BC, Ebert BL, and Fischer ES (2018). Thalidomide promotes degradation of SALL4, a transcription factor implicated in Duane radial ray syndrome. eLife 7, e38430. 10.7554/eLife.38430. 30067223 PMC6156078

[80] Meier F, Brunner A-D, Frank M, Ha A, Bludau I, Voytik E, Kaspar-Schoenefeld S, Lubeck M, Raether O, Bache N, (2020). diaPASEF: parallel accumulation–serial fragmentation combined with data-independent acquisition. Nat. Methods 17, 1229–1236. 33257825 10.1038/s41592-020-00998-0

[81] Schneider VA, Graves-Lindsay T, Howe K, Bouk N, Chen HC, Kitts PA, Murphy TD, Pruitt KD, Thibaud-Nissen F, Albracht D, (2017). Evaluation of GRCh38 and de novo haploid genome assemblies demonstrates the enduring quality of the reference assembly. Genome Res. 27, 849–864. 10.1101/gr.213611.116. 28396521 PMC5411779

[82] Lander ES, Linton LM, Birren B, Nusbaum C, Zody MC, Baldwin J, Devon K, Dewar K, Doyle M, FitzHugh W, (2001). Initial sequencing and analysis of the human genome. Nature 409, 860–921. 10.1038/35057062. 11237011

[83] Dobin A, Davis CA, Schlesinger F, Drenkow J, Zaleski C, Jha S, Batut P, Chaisson M, and Gingeras TR (2013). STAR: ultrafast universal RNA-seq aligner. Bioinformatics 29, 15–21. 10.1093/bioinformatics/bts635. 23104886 PMC3530905

[84] Li H, Handsaker B, Wysoker A, Fennell T, Ruan J, Homer N, Marth G, Abecasis G, and Durbin R; 1000 Genome Project Data Processing Subgroup (2009). The Sequence Alignment/Map format and SAMtools. Bioinformatics 25, 2078–2079. 10.1093/bioinformatics/btp352. 19505943 PMC2723002

[85] Danecek P, Bonfield JK, Liddle J, Marshall J, Ohan V, Pollard MO, Whitwham A, Keane T, McCarthy SA, Davies RM, and Li H (2021). Twelve years of SAMtools and BCFtools. GigaScience 10, giab008. 10.1093/gigascience/giab008. 33590861 PMC7931819

[86] Amemiya HM, Kundaje A, and Boyle AP (2019). The ENCODE blacklist: identification of problematic regions of the genome. Sci. Rep. 9, 9354. 10.1038/s41598-019-45839-z. 31249361 PMC6597582

[87] Robinson MD, and Oshlack A (2010). A scaling normalization method for differential expression analysis of RNA-seq data. Genome Biol. 11, R25. 10.1186/gb-2010-11-3-r25. 20196867 PMC2864565

[88] Anders S, and Huber W (2010). Differential expression analysis for sequence count data. Genome Biol. 11, R106. 10.1186/gb-2010-11-10-r106. 20979621 PMC3218662

[89] Feng J, Liu T, Qin B, Zhang Y, and Liu XS (2012). Identifying ChIP-seq enrichment using MACS. Nat. Protoc. 7, 1728–1740. 10.1038/nprot.2012.101. 22936215 PMC3868217

[90] Zhang Y, Liu T, Meyer CA, Eeckhoute J, Johnson DS, Bernstein BE, Nusbaum C, Myers RM, Brown M, Li W, and Liu XS (2008). Model-based analysis of ChIP-Seq (MACS). Genome Biol. 9, R137. 10.1186/gb-2008-9-9-r137. 18798982 PMC2592715

[91] Ross-Innes CS, Stark R, Teschendorff AE, Holmes KA, Ali HR, Dunning MJ, Brown GD, Gojis O, Ellis IO, Green AR, (2012). Differential oestrogen receptor binding is associated with clinical outcome in breast cancer. Nature 481, 389–393. 10.1038/nature10730. 22217937 PMC3272464

[92] Yu G, Wang LG, and He QY (2015). ChIPseeker: an R/Bioconductor package for ChIP peak annotation, comparison and visualization. Bioinformatics 31, 2382–2383. 10.1093/bioinformatics/btv145. 25765347

[93] Love MI, Huber W, and Anders S (2014). Moderated estimation of fold change and dispersion for RNA-seq data with DESeq2. Genome Biol. 15, 550. 10.1186/s13059-014-0550-8. 25516281 PMC4302049

[94] Stephens M (2017). False discovery rates: a new deal. Biostatistics 18, 275–294. 10.1093/biostatistics/kxw041. 27756721 PMC5379932

[95] Bailey TL, Johnson J, Grant CE, and Noble WS (2015). The MEME Suite. Nucleic Acids Res. 43, W39–W49. 10.1093/nar/gkv416. 25953851 PMC4489269

[96] Bailey TL (2021). STREME: accurate and versatile sequence motif discovery. Bioinformatics 37, 2834–2840. 10.1093/bioinformatics/btab203. 33760053 PMC8479671

[97] Bailey TL, and Grant CE (2021). SEA: simple enrichment analysis of motifs. 10.1101/2021.08.23.457422.

[98] Castro-Mondragon JA, Riudavets-Puig R, Rauluseviciute I, Lemma RB, Turchi L, Blanc-Mathieu R, Lucas J, Boddie P, Khan A, Manosalva Pé rez N, (2022). JASPAR 2022: the 9th release of the open-access database of transcription factor binding profiles. Nucleic Acids Res. 50, D165–D173. 10.1093/nar/gkab1113. 34850907 PMC8728201

[99] Li B, and Dewey CN (2011). RSEM: accurate transcript quantification from RNA-Seq data with or without a reference genome. BMC Bioinformatics 12, 323. 10.1186/1471-2105-12-323. 21816040 PMC3163565

[100] Leng N, Dawson JA, Thomson JA, Ruotti V, Rissman AI, Smits BM, Haag JD, Gould MN, Stewart RM, and Kendziorski C (2013). EBSeq: an empirical Bayes hierarchical model for inference in RNA-seq experiments. Bioinformatics 29, 1035–1043. 10.1093/bioinformatics/btt087. 23428641 PMC3624807

[101] Demichev V, Messner CB, Vernardis SI, Lilley KS, and Ralser M (2020). DIA-NN: neural networks and interference correction enable deep proteome coverage in high throughput. Nat. Methods 17, 41–44. 31768060 10.1038/s41592-019-0638-x PMC6949130

[102] R Development Core Team (2014). R: A Language and Environment for Statistical Computing (R Foundation for Statistical Computing).

[103] Ritchie ME, Phipson B, Wu D, Hu Y, Law CW, Shi W, and Smyth GK (2015). limma powers differential expression analyses for RNA-sequencing and microarray studies. Nucleic Acids Res. 43, e47. 10.1093/nar/gkv007. 25605792 PMC4402510

