# Supplementary material for: UBR5 forms ligand-dependent complexes on chromatin to regulate nuclear hormone receptor stability
Source: Mol Cell. Author manuscript; Available in PMC 2024 May 29. (PMC11134608; doi:10.1016/j.molcel.2023.06.028)
Supplement: Supplementary Material [file NIHMS1989369-supplement-Supplementary_Material.pdf]

Molecular Cell, Volume *83*

Supplemental information

UBR5 forms ligand-dependent complexes on chromatin to regulate nuclear hormone receptor stability

Jonathan M. Tsai, Jacob D. Aguirre, Yen-Der Li, Jared Brown, Vivian Focht, Lukas Kater, Georg Kempf, Brittany Sandoval, Stefan Schmitt, Justine C. Rutter, Pius Galli, Colby R. Sandate, Jevon A. Cutler, Charles Zou, Katherine A. Donovan, Ryan J. Lumpkin, Simone Cavadini, Paul M.C. Park, Quinlan Sievers, Charlie Hatton, Elizabeth Ener, Brandon D. Regalado, Micah T. Sperling, Mikołaj Słabicki, Jeonghyeon Kim, Rebecca Zon, Zinan Zhang, Peter G. Miller, Roger Belizaire, Adam S. Sperling, Eric S. Fischer, Rafael Irizarry, Scott A. Armstrong, Nicolas H. Thomä, and Benjamin L. Ebert

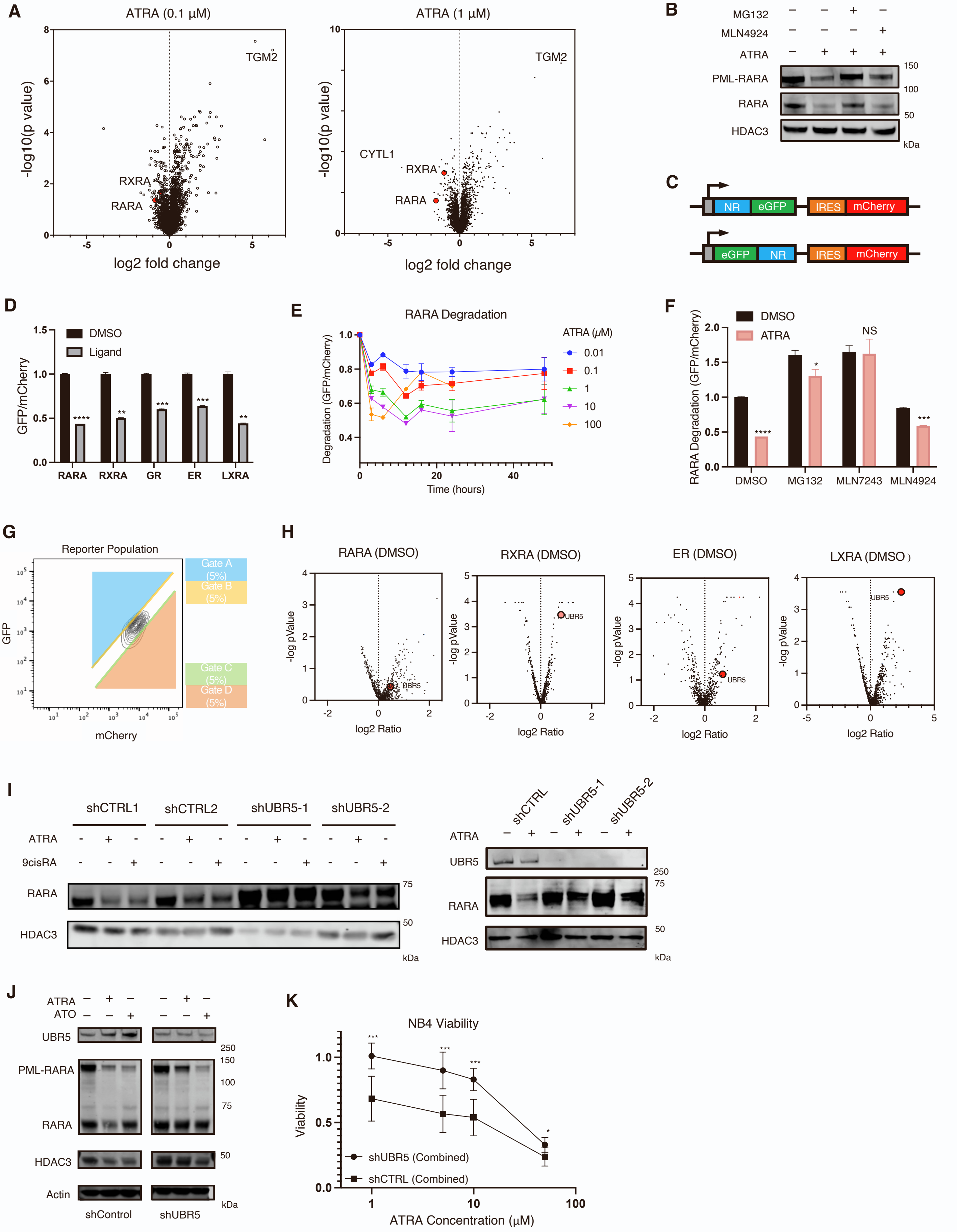

Figure S1. Ligand dependent NR degradation by UBR5, Related to Figure 1
A. Whole proteomics of NB4 cells treated with 0.1, 1 μM ATRA for 24 hours. (n=3 per condition). **B.** Western blotting of PML-RARA, RARA and HDAC3 in NB4 cells following treatment with 10 μM ATRA and proteasome inhibitor (MG132) or neddylation inhibitor (MLN4924). **C.** Schematic of nuclear hormone receptor reporters. Genes of interest are fused with GFP followed by an IRES, mCherry. **D.** NR reporter degradation following ligand (RARA:ATRA, RXRA:ATRA, GR: dexamethasone, ER: estradiol, LXRA: T0901317) treatment. **E.** RARA-GFP reporter degradation after treatment with ATRA. **F.** RARA-GFP reporter degradation after treatment with ligand, proteasome inhibitor (MG132), E1 inhibitor (MLN7243), or neddylation inhibitor (MLN4924) (n=3 two sided t-test). **G.** Schematic of CRISPR sorting screen. Top (blue) and bottom (orange) 5% gates of GFP, mCherry double positive populations are sorted and sequenced RARA reporter degradation (GFP/mCherry ratios) titrations after ATRA treatment. (n=3 two sided t-test). **H.** Volcano plots of targeted CRISPR screens highlighting UBR5 following DMSO treatment of NR fluorescent reporter U937 cell lines. **I.** Western blots of NB4 cells transduced with shRNAs against luciferase (shCTRL) or UBR5 treated +/- ATRA or 9cisRA, with adjacent replicates, blotted for RARA. **J.** Western blots of NB4 cells transduced with shRNAs against luciferase (shControl) or UBR5 treated with or without ATRA or ATO, blotted for RARA and PML-RARA. **K.** NB4 cell viability following transduction of shRNAs against luciferase or UBR5 and ATRA treatment for 3 days. Data from two shRNAs are combined. (n=4, two sided t-test) NS, not significant, $^*p < 0.05$, $^{**}p < 0.005$, $^{***}p < 0.0005$, $^{****}p < 0.00005$, error bars are SEM

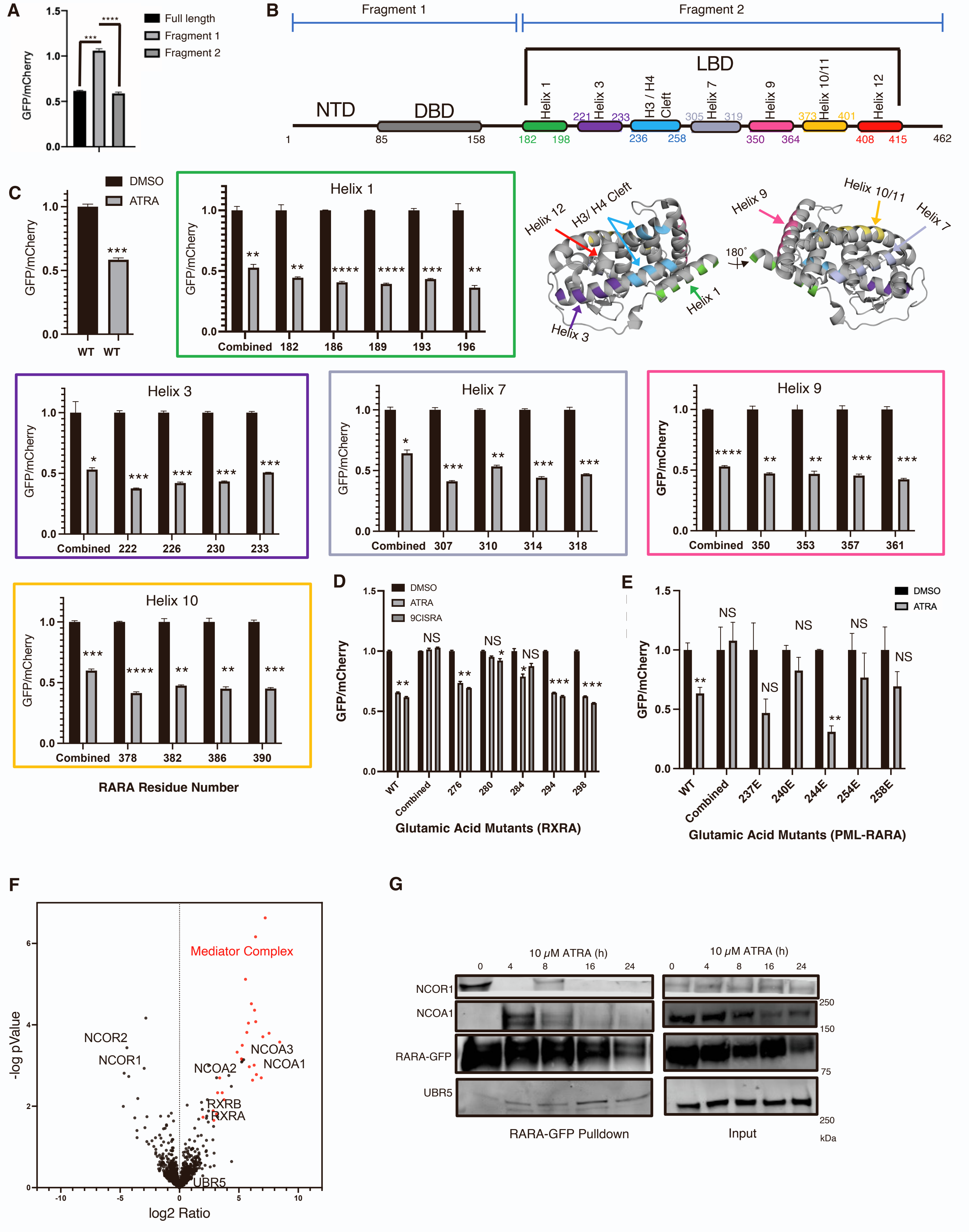

Figure S2. UBR5 competes with nuclear coactivators for the conserved hydrophobic cleft of NRs, Related to Figure 2

A. Degradation of subdomains of RARA reporters after treatment with ATRA. Fragment boundaries are denoted above the schematic in panel B. **B.** Schematic of RARA domain architecture; NTD = N-terminal domain, DBD = DNA binding domain, LBD = ligand binding domain. Interrogated helices and cleft are denoted by colors and indicated residues. **C.** Degradation (GFP/mCherry ratio) of RARA reporters containing denoted alanine residue substitutions following ATRA treatment. The structure of RARA above illustrates the position of the surface residues substituted in the experiment. **D.** Degradation (GFP/mCherry ratio) of RXRA reporters containing single point glutamate substitutions within the LBD after treatment with 9cisRA or ATRA. **E.** Degradation (GFP/mCherry ratio) of PML-RARA reporters containing single point glutamate substitutions within the LBD following ATRA treatment. (n=3, two sided t-test) NS = not significant, * p<0.05, ** p<0.005, *** p<0.0005, **** p<0.00005). **F.** Volcano plot of RARA IPMS following 8 hours of ATRA treatment. Members of the mediator complex are colored in red. **G.** Western blots of RARA-GFP co-immunoprecipitations from U937 RARA-GFP reporter cells treated with ATRA/MG132 for 0, 4, 8, 16, 24 hours, and blotted with antibodies specific for NCOR1, NCOA1, GFP, and UBR5 as indicated.

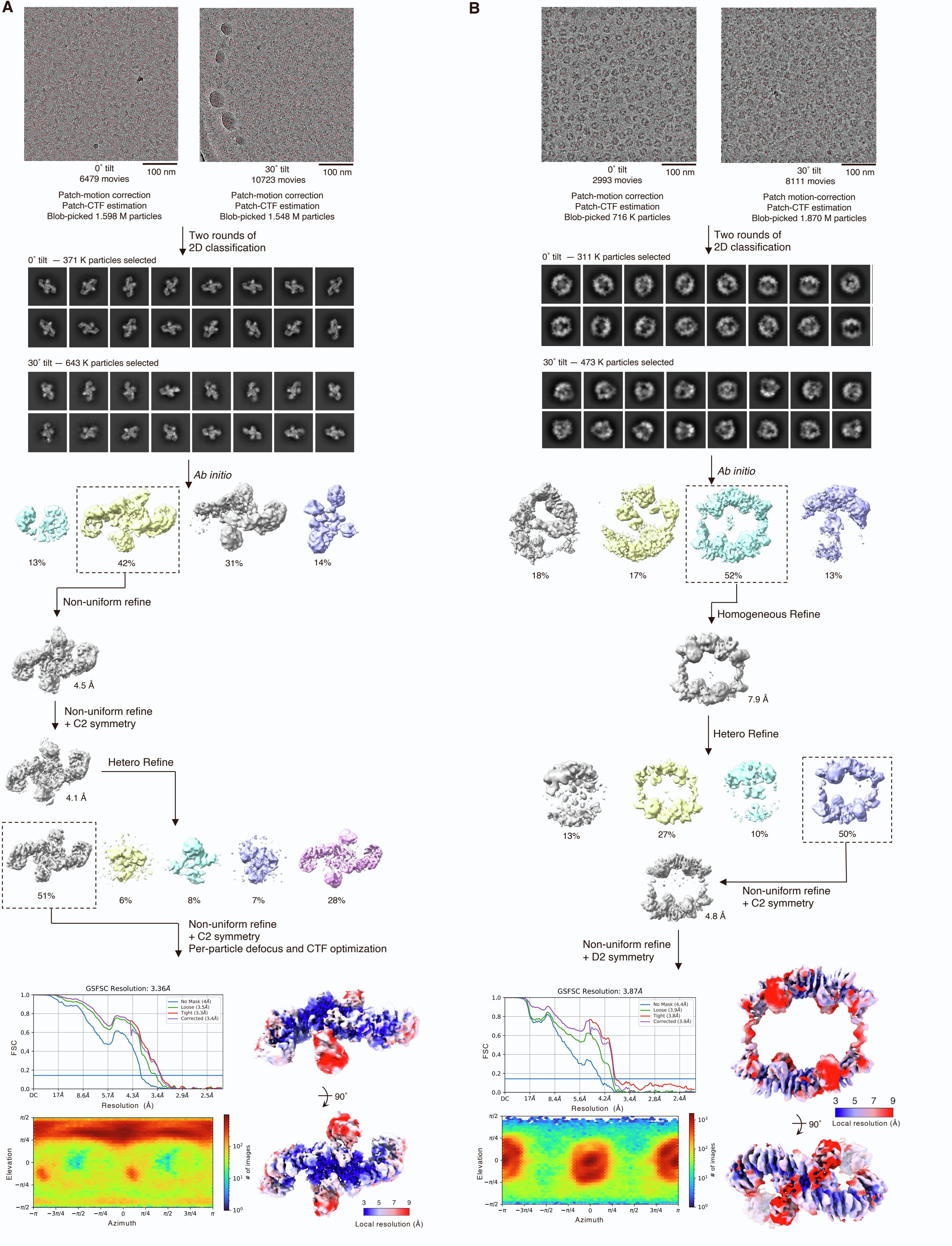

Figure S3. Cryo-EM processing of UBR5 tetramer and dimer datasets, Related to Figure 3
Data processing pipelines for **A.** UBR5 dimer and **B.** UBR5 tetramer cryo-EM density maps. All data were processed entirely in cryoSPARC v3. Representative motion-corrected micrographs are shown at the top. Stage-tilted (30˚) and non-tilted (0˚) datasets were imported into cryoSPARC v3.3 and motion correction and CTF estimation performed in patch mode. Subsequent ab-initio, classification and refinement steps are shown. The final models are presented in 2 orientations and colored by local resolution (see legends). Viewing angle distribution maps and Gold-standard fourier shell correlation (GSFSC) curves are shown after FSC mask auto-tightening for the final density maps.

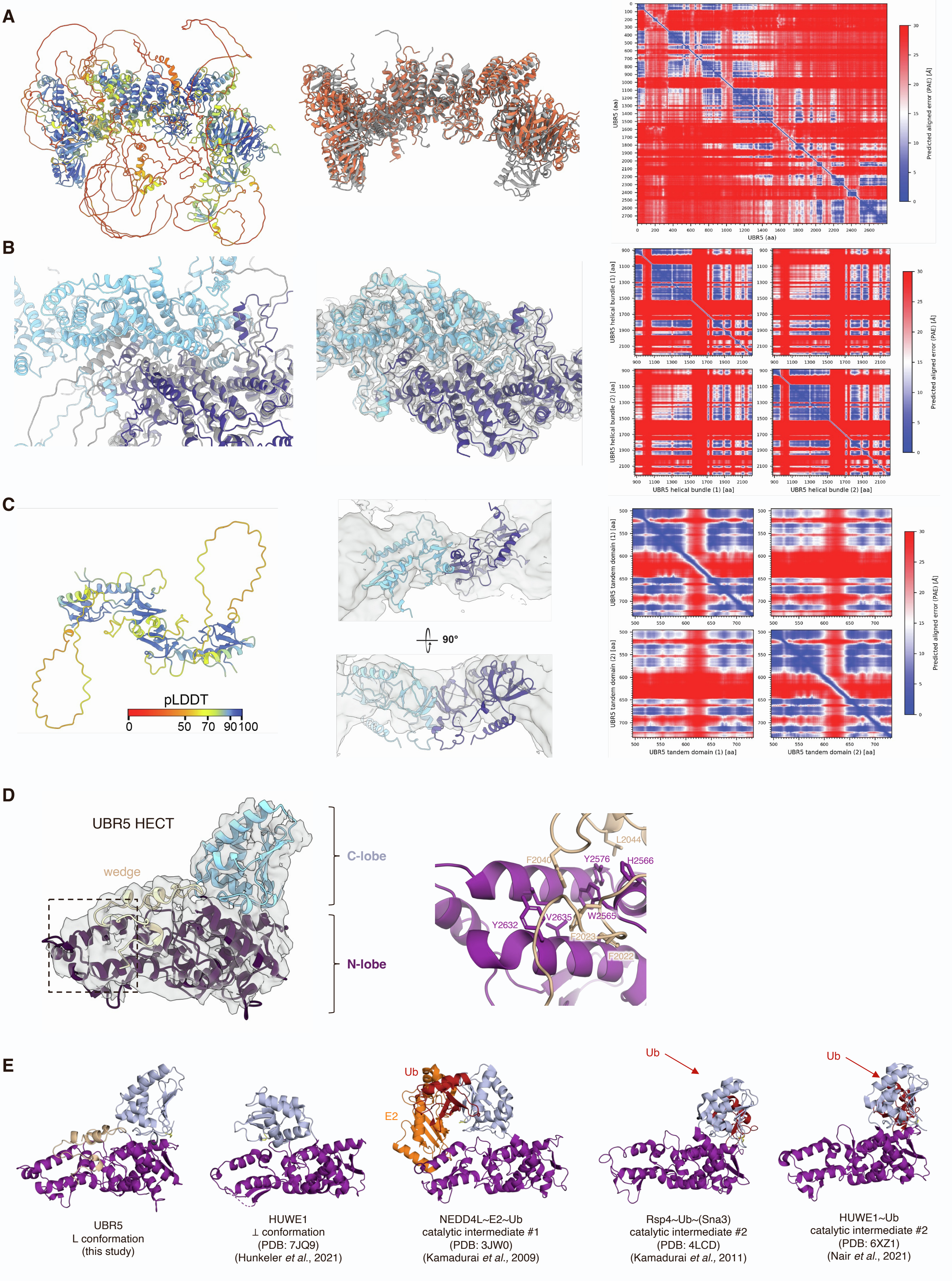

Figure S4. Model building of UBR5 structure, Related to Figure 3

A. AlphaFold prediction of full-length UBR5. Left panel, model colored by pLDDT values (color key in C). middle panel, superposition of predicted model and model refined against the cryo-EM map (r.m.s.d. 8.7 Å). Right panel, predicted aligned error (PAE) plot from AlphaFold. **B.** Prediction of UBR5 helical bundle dimerization interface with AlphaFold-multimer using fitted cryo-EM monomer model as template. left panel, superposition of predicted monomer model (gray) and predicted dimer model (blue) to illustrate local conformational differences. middle panel, map-fit of refined cryo-EM model using predicted dimer as reference. right panel, PAE plot from AlphaFold-multimer. Each subunit combination is plotted separately for clarity. **C.** Prediction of UBR5 tandem domain (aa 495-732) with AlphaFold-multimer. left panel, predicted dimer model colored by pLDDT. Middle panel, model (aa 495-598, 640-732) refined against the cryo-EM map (gray). Right panel, PAE plot from AlphaFold-multimer. **D.** EM density and fitted model for the UBR5 HECT domain, showing an L conformation that is induced by the "wedge" (coloured beige). Specific hydrophobic side chains that stabilize the wedge interaction with the HECT N-lobe are shown on the right panel zoomed in. **E.** Comparison of UBR5 to other available HECT structures exemplifying conformational variability observed in different intermediate states.

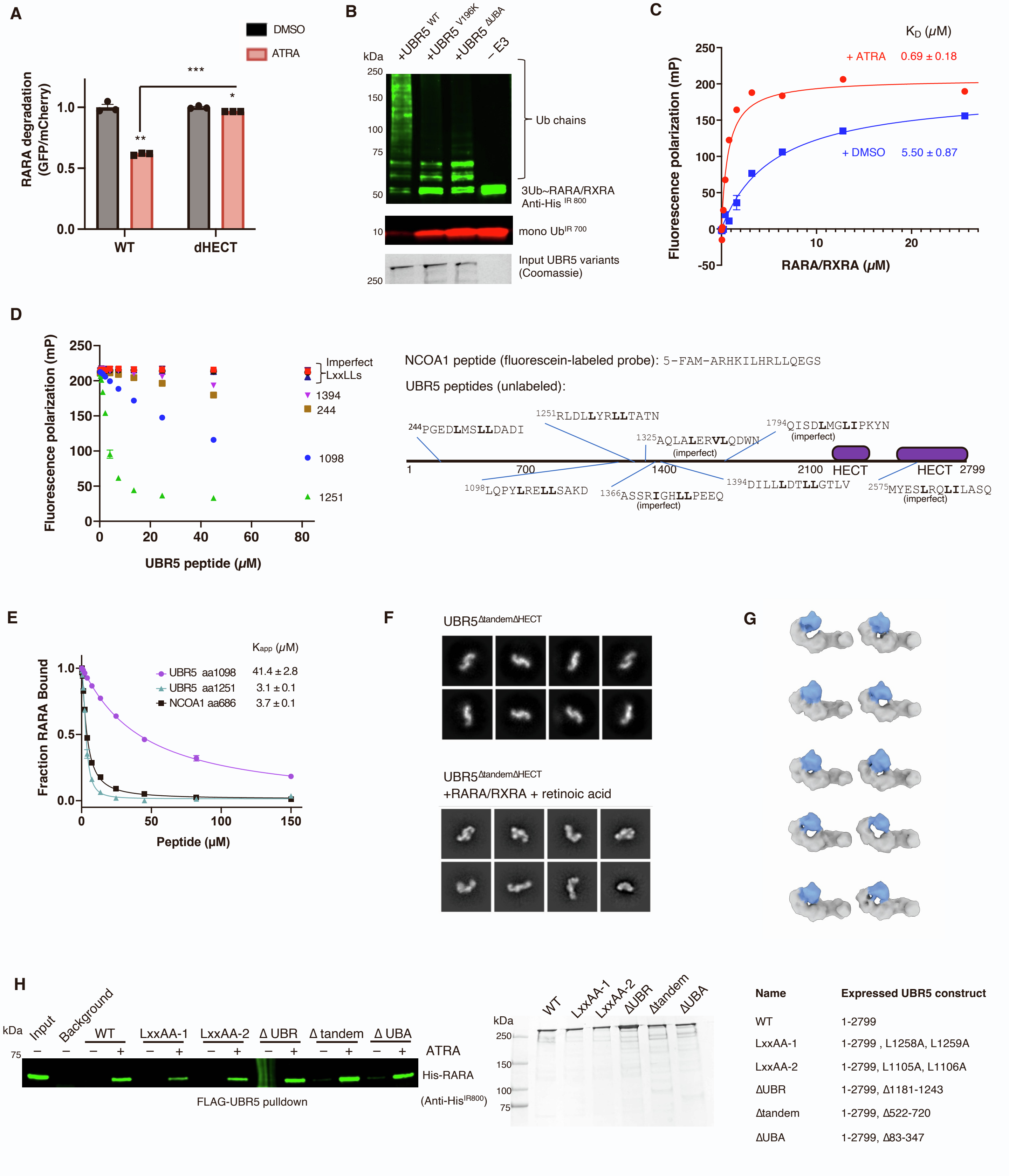

Figure S5. Interaction of UBR5 with NRs and LxxLL motifs, Related to Figure 3

A. RARA reporter degradation in HEK293 cells with CRISPR-edited UBR5 deficient of its HECT domain in the presence or absence of ATRA (n=3 two-sided t test). **B.** Ubiquitylation assay of a pseudo-primed RARA/RXRA substrate, generated as His-3Ub~RARA and His-3Ub~RXRA linear fusions in E.coli. Wild type UBR5 can rapidly branch and extend Ub chains on the substrate, however indicated mutants of the UBA domain severely hinder chain extension. **C.** Fluorescence polarization (FP) experiment showing forward titration of RARA/RXRA into a fluorescein-labelled NCOA1 peptide (residues 686-698). Complex formation is measured by an increase of mP value and was fit to a single binding event to determine binding constant (K_D). **D.** Reverse titration FP experiment, to determine if LxxLL motifs in UBR5 can bind RARA/RXRA. Synthetic UBR5 peptides (exact sequences listed on the left) were titrated into a pre-formed complex of fluorescein-labelled NCOA1 peptide (residues 686-698) and RARA/RXRA. Binding of UBR5 peptides to RARA/RXRA is reported by out-competition of the fluorescein-labelled peptide and observed as reduced mP value. The 2 best peptide hits were repeated in a finer titration and compared to unlabeled NCOA1 peptide in panel E. Experiments were conducted as duplicates. **E.** Fluorescence polarization experiments depicting competitive reverse titration with indicated peptides against a pre-formed complex of RARA/RXRA with a fluorescein-labelled peptide of NCOA1 (residues 686-698) in the presence of ATRA. Titrations were performed in duplicate and binding constants determined after converting fluorescence polarization values to fraction bound of original complex. **F.** Representative 2D class averages from negative stain datasets which produced the models presented in main Figure 3F. 2D classification was performed in cryoSPARC v4, with each class reconstructed from 500-700 particles. **G.** 3D classification of particles which make up the consensus map shown in Figure 3F. Classification was performed in cryoSPARC v4 with 10 output classes composed of 500-700 particles each. The additional density region corresponding to RARA/RXRA is colored blue, **H.** Western blot showing *in vitro* pull-down of purified His-tagged RARA/RXRA heterodimer by FLAG-UBR5 and mutants thereof (listed in G). In all cases, FLAG M2 beads were saturated with indicated UBR5 mutant, followed by addition of His-RARA/RXRA heterodimer in the presence of 50 μM ATRA or DMSO. A coomassie-stained gel of the purified UBR5 variants and a description of expressed constructs used in this experiment is shown beside.

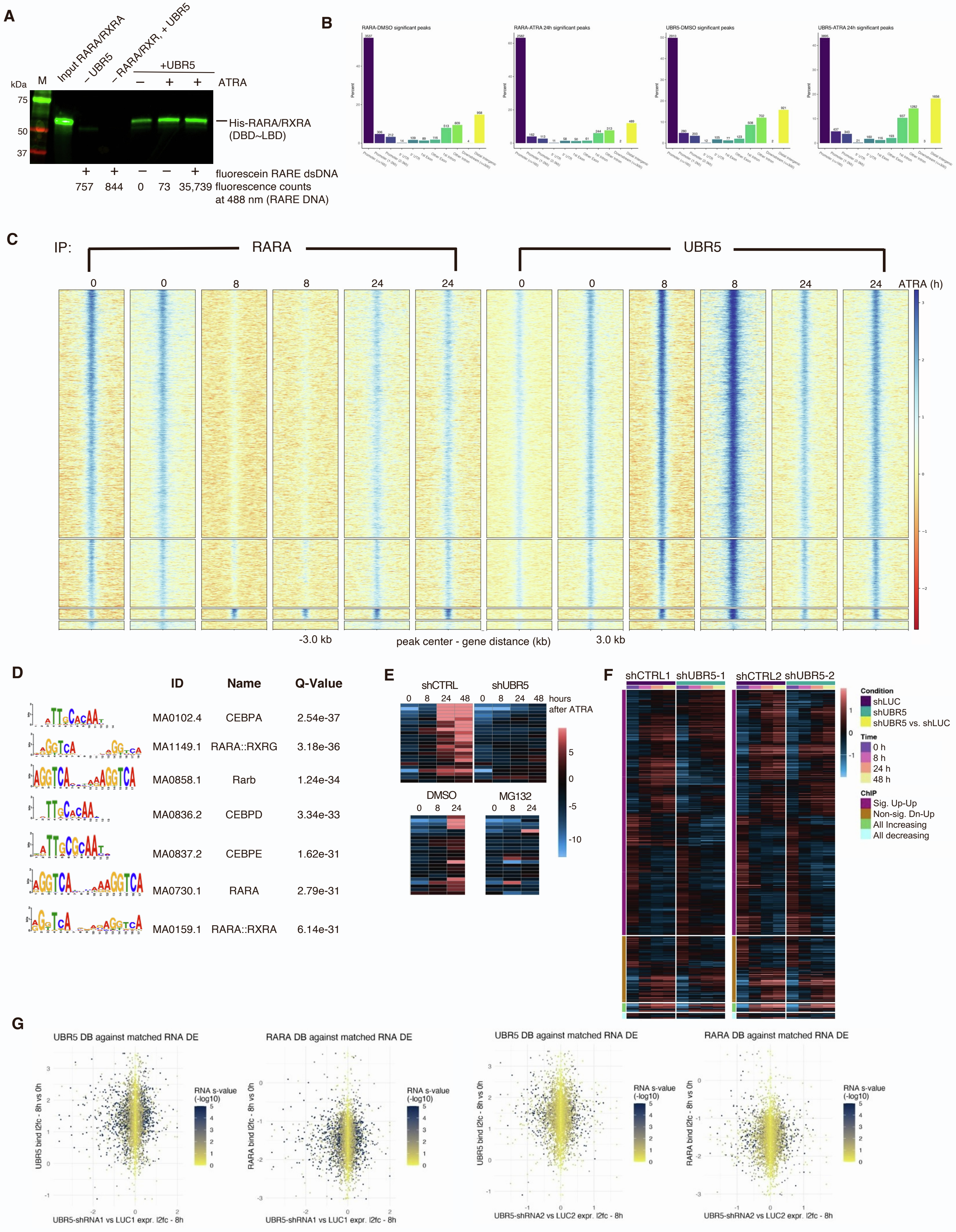

Figure S6. UBR5 targets RARA on chromatin and regulates transcription, Related to Figure 4

A. Western blot (anti-His) showing *in vitro* FLAG-UBR5 pulldown of purified His-tagged RARA/RXRA heterodimer, showing that a fluorescein-labelled 24-mer dsDNA containing a DR5 Retinoic Acid Response Element (RARE) sequence is readily incorporated into a UBR5~RARA/RXRA complex in the presence of ATRA. Immediately before preparing samples for SDS-PAGE, fluorescence counts were measured at 488 nm to assess dsDNA incorporation. Fluorescence counts were normalized to the –dsDNA, DMSO condition as a baseline. **B.** Distribution of differentially bound peaks in ChIP-seq experiments pulling down (in order) RARA (DMSO treatment), RARA (ATRA treatment), UBR5 (DMSO treatment), UBR5 (ATRA treatment). **C.** Tornado plots of chromatin-immunoprecipitation sequencing (ChIP-seq) targeting RARA or UBR5 with or without ATRA in NB4 cells, clustered by change following ATRA treatment, in duplicate. Plots are centered around peak center and represent log2 fold change over input (n=2). **D.** Motif enrichment analysis against the Jasper 2022 Vertebrate database of UBR5 peaks gained following ATRA treatment. **E.** RNA sequencing of NB4 cells transduced with shRNAs or MG132 against luciferase or UBR5, treated with ATRA for 0, 8, 24 and 48 hours. Genes were filtered for reported ATRA-induced genes. Heat map represents log-scale Pearson residuals. (n=2) **F.** Global RNA sequencing of NB4s transduced with shRNAs against luciferase or UBR5, treated with ATRA for 0, 8, 24, 48 hours. Genes displayed were filtered by genes that were significantly regulated by ATRA (s-value <=0.01) between 0 and 24h in both shCTRLs, had an annotated RARA peak, and subsetted such that the nearest RARA peak falls into one of the three differentially bound groups in the RARA ChIPseq (Figure 4A). (n=2) **G.** Global RNA sequencing analysis comparing NB4s transduced with shRNAs against UBR5 to luciferase, treated with ATRA for 8 hours.

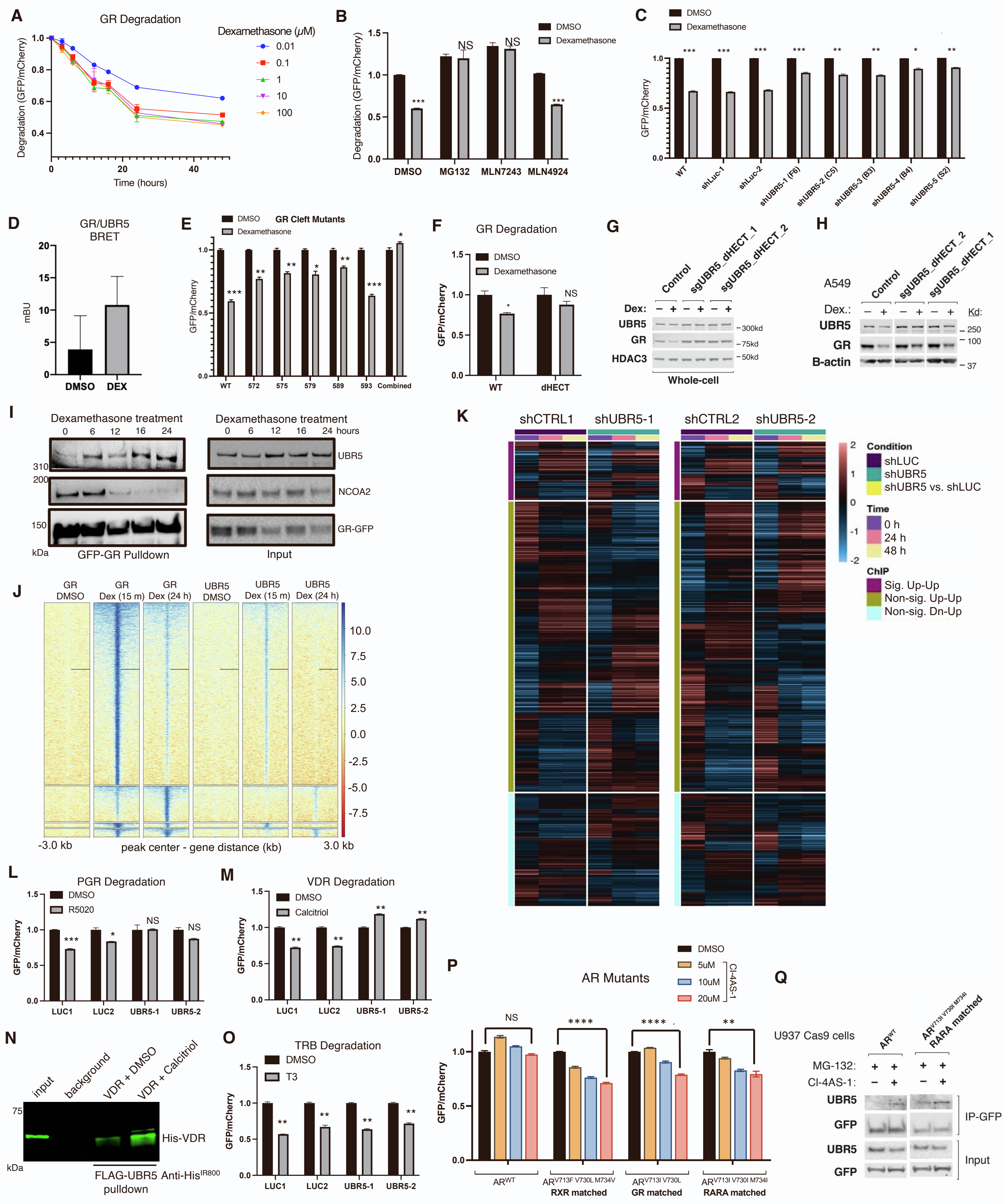

Figure S7. UBR5 regulates a greater subset of NRs through a common degron, Related to Figure 5

A. GR reporter degradation (GFP/mCherry ratios) titrations after dexamethasone treatment. (n=3 two sided t-test) **B.** GFP-GR reporter degradation after treatment with dexamethasone and proteasome inhibitor (MG132), E1 inhibitor (MLN7243), or neddylation inhibitor (MLN4924) (n=3 two sided t-test). **C.** GR reporter degradation (GFP/mCherry) following transduction of shRNAs against luciferase or UBR5 (5x shRNAs) and treatment with dexamethasone (n=3 two sided t-test). **D.** Bioluminescence Resonance Energy Transfer (BRET) assay of GR, treated +/- dexamethasone. (n=3) **E.** Degradation (GFP/mCherry ratio) of GR reporters containing single point mutations within the LBD after treatment with dexamethasone. (n=3 two sided t-test) **F.** GR Reporter degradation in HEK293Ts with sgRNAs targeting the UBR5 HECT domain transduced with GFP-GR reporters and treated with dexamethasone. (n=3 two sided t-test). **G.** Western blots of U937 cells transduced with sgRNAs targeting the UBR5 HECT domain +/- dexamethasone. **H.** Western blots of A549 cells transduced with sgRNAs targeting the UBR5 HECT domain +/- dexamethasone. **I.** Western blots of GR co-immunoprecipitations from U937 GFP-GR reporter cells following dexamethasone treatments. **J.** Tornado plots of a second replicate of ChIP-seq targeting GR or UBR5 +/- dexamethasone in A549 cells. Specific analysis of the loci with greatest GR recruitment, defined as regions with peak loss from 15 minutes to 24 hours of dexamethasone treatment, revealed an even more robust UBR5 association. UBR5 is similarly absent at baseline and eliminated after 24 hours, emulating GR. Plots are centered around peak center and represent log2 fold change over input. **K.** Global RNA sequencing of A549s transduced with shRNAs against luciferase or UBR5, treated with dexamethasone for 0, 24, 48 hours. Genes displayed were filtered by genes that were significantly regulated by dexamethasone (s-value <=0.01) between 0 and 24h in both shCTRLs, had an annotated GR peak, and subsetted such that the nearest GR peak falls into one of the three differentially bound groups in the GR ChIPseq (Figure 5C). (n=2) **L.** Degradation (GFP/mCherry) of progesterone receptor (PGR) in U937s transduced with shRNAs targeting luciferase or UBR5 following treatment with R5020. (n=3 two sided t-test) **M.** Degradation (GFP/mCherry) of vitamin-D receptor (VDR) in U937s transduced with shRNAs targeting luciferase or UBR5 following treatment with calcitriol (n=3 two sided t-test) **N.** Western blot showing *in vitro* FLAG-UBR5 pulldown of purified His-tagged VDR or in the presence of calcitriol. **O.** Degradation (GFP/mCherry) of thyroid hormone receptor beta (THRB) in U937s transduced with shRNAs targeting luciferase or UBR5 following treatment with levothyroxine (T3). (n=3 two sided t-test) **P.** Degradation (GFP/mCherry) of fluorescent Androgen Receptor (AR) reporters with substitutions to residues in the hydrophobic cleft swapped to match those residues found in RXRA, GR, and RARA treated with different amounts of CI-4AS-1. (n=3 two sided t-test) **Q.** Co-immunoprecipitation blots of AR wild-type and RARA hydrophobic cleft mutants construct +/- CI-4AS-1 and MG132. (NS = not significant, * p<0.05, ** p<0.005, *** p<0.0005, **** p<0.00005)

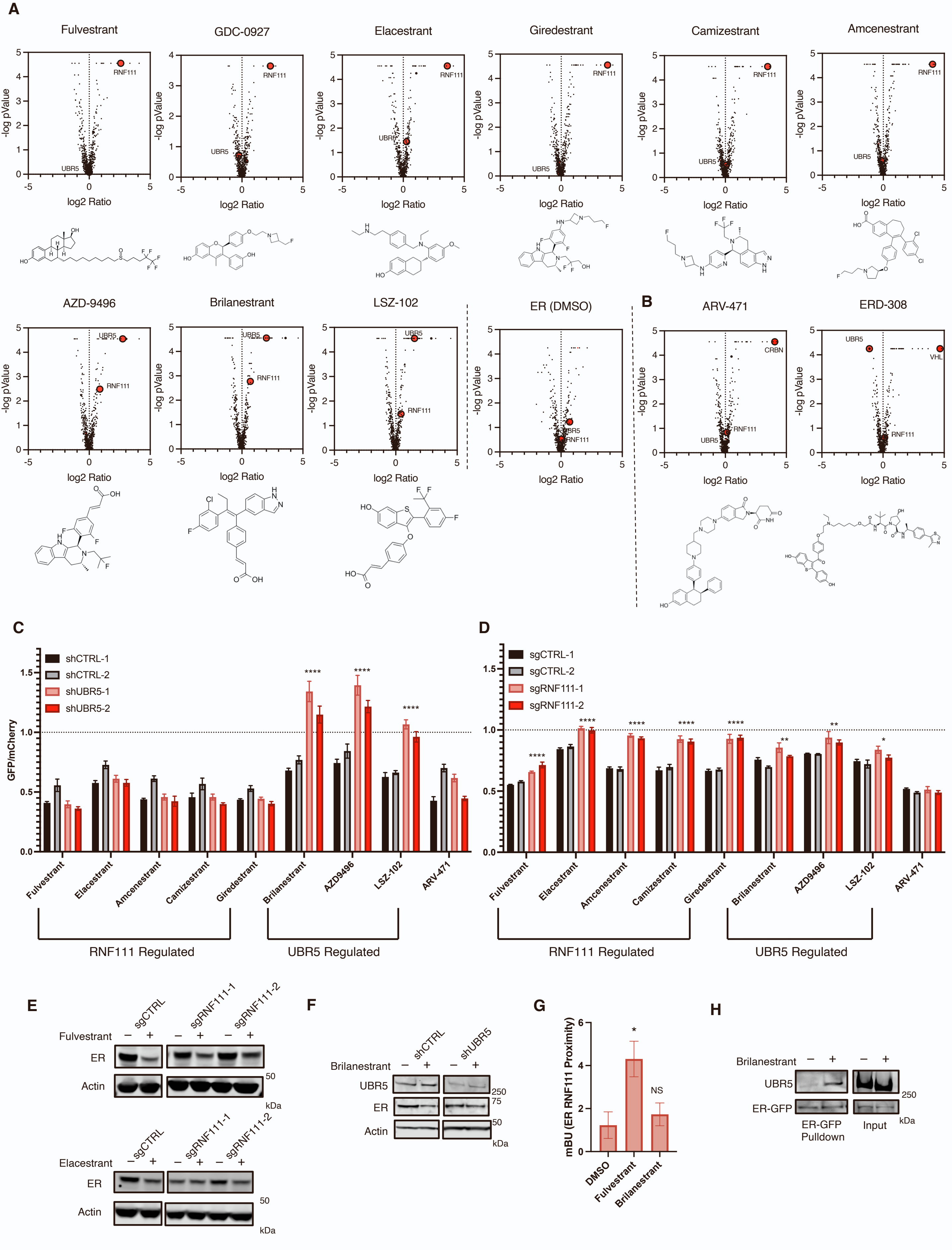

Figure S8. Non-endogenous ligands recruit different E3 ligases to induce ER degradation, Related to Figure 6
(A, B) Volcano plots of targeted CRISPR screens highlighting enrichment of UBR5 or RNF111 following SERD treatment of ER fluorescent reporter K562 cell lines. UBR5, RNF111, CRBN, VHL are highlighted in red. **(C)** Degradation (GFP/mCherry) of ER-GFP in K562 reporter cell lines treated with shRNAs targeting UBR5 and the specified SERDs or ER PROTACs. **(D)** Degradation (GFP/mCherry) of ER-GFP in K562 reporter cell lines treated with sgRNAs targeting RNF111 and the specified SERDs or ER PROTACs. **(E)** Western blots of T47D cells transduced with sgRNAs against RNF111 treated with or without fulvestrant or elacestrant. **(F)** Western blots of T47D cells transduced with shRNAs against luciferase (shControl) or UBR5 treated with or without Brilanestrant. **(G)** Bioluminescence Resonance Energy Transfer (BRET) assay of ER, treated +/- fulvestrant or brilanestrant. (n=6 two sided t-test) **(H)** Western blots of ER co-immunoprecipitations from K562 ER-GFP reporter cells treated with brilanestrant and MG132 for 16 hours, and blotted with antibodies specific for GFP, and UBR5 as indicated. NS, not significant, *p < 0.05, **p < 0.005, ***p < 0.0005, ****p < 0.00005, error bars are SEM

Supplemental Table S1: Cryo-EM data collection, refinement and validation statistics, related to Figure 3

	UBR5 tetramer (EMDB-17540) (PDB: 8P83)	UBR5 dimer (EMDB-17539) (PDB: 8P82)
**Data collection and processing**		
Magnification	59,000x	59,000x
Voltage (kV)	300	300
Electron exposure (e–/Å^2)	50[a]	50[a]
Defocus range (μm)	-1.0 to -2.2	-1.0 to -2.2
Pixel size (Å)	1.118	1.118
Symmetry imposed	D2	C2
Initial particle images (no.)	3,146,000	2,586,000
Final particle images (no.)	393,327	399,325
Map resolution (Å)	3.87	3.36
FSC threshold	0.143	0.143
Map resolution range (Å)	3-9Å	3-9Å
**Refinement**		
Initial model used (PDB code)	UBR5 dimer	AlphaFold prediction
Model resolution (Å)	3.87	3.36
FSC threshold	0.143	0.143
Model resolution range (Å)		
Map sharpening *B* factor (Å^2)	N.A.[b]	N.A.[b]
Model composition		
Non-hydrogen atoms	36,240	25,156
Protein residues	7,148	3,192
Ligands	0	0
B factors (Å^2)		
Protein	341.82	276.04
Ligand	N.A.	N.A.
R.m.s. deviations		
Bond lengths (Å)	0.006	0.013
Bond angles (°)	1.051	1.314
Validation		
MolProbity score	0.81	1.10
Clashscore	0.86	1.15
Poor rotamers (%)	N.A.	0.07
Ramachandran plot		
Favored (%)	97.79	96.02
Allowed (%)	2.04	3.78
Disallowed (%)	0.17	0.19

[a] Data collected at 30˚ stage tilt were collected with an increased dose of 55 e^-/A^2

[b] The map was locally sharpened using LocScale

Supplemental Table S2: Clinical Development of Selective Estrogen Receptor Degraders (SERDs), related to Figure 6

Compound	Side Chain	E3 Ligase	Clinical Development Status for HR+ HER2- Advanced Breast Cancer Treatment
Fulvestrant	Long Alkyl	RNF111	FDA approved for first-line and second-line treatments[1,2]
Elacestrant (RAD1901)	Basic Amino	RNF111	FDA approved for second-line, ESR1-mutated disease treatment[3,4]; Ongoing phase III trial for first-line treatment (NCT05512364)
Camizestrant (AZD9833)	Basic Amino	RNF111	Ongoing Phase III trial for first-line treatment (NCT04711252, NCT04964934); Successful phase II trial for second-line treatment (NCT04214288)[5]
Giredestrant (GDC-9545)	Basic Amino	RNF111	Ongoing phase III trial for first-line treatment (NCT04546009); Failed primary endpoint in phase II trial for second-line treatment (NCT04576455)[6]
Amcenestrant (SAR439859)	Basic Amino	RNF111	Failed primary endpoint phase III trial for first-line treatment (NCT04478266)[7]; Failed primary endpoint in phase II trial for second-line treatment (NCT04059484)[8] [Discontinued, 2022]
GDC-0927	Basic Amino	RNF111	Completed phase I trial (NCT02316509)[9] [Discontinued, 2017[10]]
Brilanestrant (GDC-0810)	Acrylic Acid	UBR5	Terminated phase I/II trials (NCT02569801, NCT01823835)[11] [Discontinued, 2017[10]]
AZD9496	Acrylic Acid	UBR5	Completed phase I trial (NCT02248090, NCT03236974)[12] [Discontinued, 2021[10]]
LSZ-102	Acrylic Acid	UBR5	Terminated phase I/Ib trial (NCT02734615)[13] [Discontinued, 2021[10]]
ARV-471	PROTAC	CRBN	Ongoing phase III monotherapy trial for second-line treatment (NCT05654623). Completed phase I/II monotherapy trial (NCT04072952)[14]. Ongoing phase I and II combo-therapy trials (NCT05501769, NCT04072952, NCT05573555, NCT05548127)
ERD-308	PROTAC	VHL	Pre-clinical stage

Supplementary Reference

1. Nathan, M.R., and Schmid, P. (2017). A review of fulvestrant in breast cancer. Oncology and therapy *5*, 17-29.
2. Carlson, R.W. (2005). The history and mechanism of action of fulvestrant. Clinical breast cancer *6*, S5-S8
3. Bardia, A., Aftimos, P., Bihani, T., Anderson-Villaluz, A.T., Jung, J., Conlan, M.G., and Kaklamani, V.G. (2019). EMERALD: Phase III trial of elacestrant (RAD1901) vs endocrine therapy for previously treated ER+ advanced breast cancer. Future oncology *15*, 3209-3218.
4. Varella, L., and Cristofanilli, M. (2023). Evaluating Elacestrant in the Management of ER-Positive, HER2-Negative Advanced Breast Cancer: Evidence to Date. OncoTargets and Therapy, 189-196.
5. Oliveira, M., Pominchuk, D., Nowecki, Z., Hamilton, E., Kulyaba, Y., Andabekov, T., Hotko, Y., Melkadze, T., Nemsadze, G., and Neven, P. (2022). Camizestrant, a Next-Generation Oral SERD vs. Fulvestrant in Post-Menopausal Women with Advanced ER-Positive HER2− Negative Breast Cancer: Results of the Randomized, Multi-Dose Phase 2 SERENA-2 Trial.
6. Jimenez, M.M., Lim, E., Mac Gregor, M.C., Bardia, A., Wu, J., Zhang, Q., Nowecki, Z., Cruz, F., Safin, R., and Kim, S. (2022). 211MO Giredestrant (GDC-9545) vs physician choice of endocrine monotherapy (PCET) in patients (pts) with ER+, HER2–locally advanced/metastatic breast cancer (LA/mBC): primary analysis of the phase II, randomised, open-label acelERA BC study. Annals of Oncology *33*, S633-S634.
7. Bardia, A., Cortes, J., Hurvitz, S.A., Delaloge, S., Iwata, H., Shao, Z.-M., Kanagavel, D., Cohen, P., Liu, Q., and Cartot-Cotton, S. (2022). AMEERA-5: a randomized, double-blind phase 3 study of amcenestrant plus palbociclib versus letrozole plus palbociclib for previously untreated ER+/HER2–advanced breast cancer. Therapeutic Advances in Medical Oncology *14*, 17588359221083956.
8. Tolaney, S., Chan, A., Petrakova, K., Delaloge, S., Campone, M., Iwata, H., Peddi, P., Kaufman, P., De Kermadec, E., and Liu, Q. (2022). 212MO AMEERA-3, a phase II study of amcenestrant (AMC) versus endocrine treatment of physician's choice (TPC) in patients (pts) with endocrine-resistant ER+/HER2− advanced breast cancer (aBC). Annals of Oncology *33*, S634-S635.
9. Dickler, M.N., Villanueva, R., Perez Fidalgo, J., Mayer, I.A., Boni, V., Winer, E.P., Hamilton, E.P., Bellet, M., Urruticoechea, A., and Gonzalez-Martin, A. (2018). Abstract PD5-10: A first-in-human phase I study to evaluate the oral selective estrogen receptor degrader (SERD), GDC-0927, in postmenopausal women with estrogen receptor positive (ER+) HER2-negative metastatic breast cancer (BC). Cancer research *78*, PD5-10-PD15-10.
10. Chen, Y.-C., Yu, J., Metcalfe, C., De Bruyn, T., Gelzleichter, T., Malhi, V., Perez-Moreno, P.D., and Wang, X. (2022). Latest generation estrogen receptor degraders for the treatment of hormone receptor-positive breast cancer. Expert Opinion on Investigational Drugs *31*, 515-529.
11. Bardia, A., Mayer, I., Winer, E., Linden, H.M., Ma, C.X., Parker, B.A., Bellet, M., Arteaga, C.L., Cheeti, S., Gates, M., et al. (2023b). The oral selective estrogen receptor degrader GDC-0810 (ARN-810) in postmenopausal women with hormone receptor-positive HER2-negative (HR + /HER2 −) advanced/metastatic breast cancer. Breast Cancer Research and Treatment *197*, 319-331. 10.1007/s10549-022-06797-9.
12. Hamilton, E.P., Patel, M.R., Armstrong, A.C., Baird, R.D., Jhaveri, K., Hoch, M., Klinowska, T., Lindemann, J.P., Morgan, S.R., and Schiavon, G. (2018). A First-in-Human Study of the New Oral Selective Estrogen Receptor Degrader AZD9496 for ER+/HER2− Advanced Breast CancerPhase I Study of Oral SERD AZD9496. Clinical Cancer Research *24*, 3510-3518.
13. Jhaveri, K., Juric, D., Yap, Y., Cresta, S., Layman, R.M., Duhoux, F.P., Terret, C., Takahashi, S., Huober, J., and Kundamal, N. (2021). A phase I study of LSZ102, an oral selective estrogen receptor degrader, with or without ribociclib or alpelisib, in patients with estrogen receptor-positive breast cancer. Clinical Cancer Research, 1-12
14. Schott, A.F., Hurvitz, S., Ma, C., Hamilton, E., Nanda, R., Zahrah, G., Hunter, N., Tan, A.R., Telli, M., and Mesias, J.A. (2023). Abstract GS3-03: GS3-03 ARV-471, a PROTAC® estrogen receptor (ER) degrader in advanced ER-positive/human epidermal growth factor receptor 2 (HER2)-negative breast cancer: phase 2 expansion (VERITAC) of a phase 1/2 study. Cancer Research *83*, GS3-03-GS03-03.
